# Multielement Characterization and Antioxidant Activity of Italian Extra-Virgin Olive Oils

**DOI:** 10.3389/fchem.2021.769620

**Published:** 2021-11-16

**Authors:** Maria Luisa Astolfi, Federico Marini, Maria Agostina Frezzini, Lorenzo Massimi, Anna Laura Capriotti, Carmela Maria Montone, Silvia Canepari

**Affiliations:** ^1^ Department of Chemistry, Sapienza University of Rome, Rome, Italy; ^2^ Department of Environmental Biology, Sapienza University of Rome, Rome, Italy

**Keywords:** authenticity, chemometrics, inductively coupled plasma mass spectrometry, olive oil, statistical analysis, trace elements, traceability

## Abstract

Food product safety and quality are closely related to the elemental composition of food. This study combined multielement analysis and chemometric tools to characterize 237 extra-virgin olive oil (EVOO) samples from 15 regions of Italy, and to verify the possibility of discriminating them according to different quality factors, such as varietal or geographical origin or whether they were organically or traditionally produced. Some elements have antioxidant properties, while others are toxic to humans or can promote oxidative degradation of EVOO samples. In particular, the antioxidant activity of oils’ hydrophilic fraction was estimated and the concentrations of 45 elements were determined by inductively coupled plasma mass spectrometry (ICP-MS). At first, univariate and multivariate analyses of variance were used to compare the element concentrations, and statistically significant differences were found among samples from different regions. Successively, discriminant classification approaches were used to build a model for EVOO authentication, considering, in turn, various possible categorizations. The results have indicated that chemometric methods coupled with ICP-MS have the potential to discriminate and characterize the different types of EVOO, and to provide “typical” elemental fingerprints of the various categories of samples.

## 1 Introduction

The elemental composition of foods is of toxicological and nutritional interest and can be considered an important quality parameter (Astolfi et al., 2021a; 2020a; 2020b). In particular, the concentrations of trace elements in extra-virgin olive oil (EVOO) are also one of the criteria for the assessment of the quality regarding storable period and freshness (Choe and Min, 2006). In fact, some elements, such as Ca, Co, Cu, Fe, Mg, Mn, Ni, and Sn, can promote the oxidative degradation of this important component of the Mediterranean diet appreciated among consumers for its nutritional properties and specific flavor (Choe and Min, 2006). Other elements (such as As, Cd, Cr, Cu, Hg, and Pb) present in EVOO are of great concern because they are toxic and potentially carcinogenic to humans even at low concentration (Tchounwou et al., 2012). The International Olive Council has established, as a quality criterion, a maximum residue level (MRL) for the content of As, Cu, Pb (0.1 mg kg^−1^), and Fe (3 mg kg^−1^) in olive oils and olive–pomace oils (International Olive Council, 2019), and the maximum levels of Cu and Fe in other vegetable oils have been also legislated (Codex Stan 33-1981, 2021), varying from 0.1 up to 5.0 mg kg^−1^. Recently, element determination in EVOO samples has gained importance for oil geographical traceability and authentication (Cordella et al., 2002; Dugo et al., 2004; Benincasa et al., 2007; Cabrera Vique et al., 2012; Camin et al., 2010; Beltran et al., 2015; Bajoub et al., 2018; Aceto et al., 2019; Damak et al., 2019; Zaroual et al., 2021). In particular, elements are useful in the characterization of protected designations of origin (PDOs) or protected geographical indications (PGIs) (European Union (EU), 2012), and they can also contribute to determine EVOO geographical provenance of non-PDO oils (Beltran et al., 2015; Aceto et al., 2019). In fact, the presence of metals in EVOO varies according to their origin and can be due to natural contamination from the soil, environment, fertilizers, and genotype of the plant or to the production process and contact with storage materials (Zeiner et al., 2005; Chatzistathis et al., 2009; Kabata-Pendias, 2010; Lepri et al., 2011; Yaşar et al., 2012; Bakircioglu et al., 2013). A suitable statistical treatment of trace element data could allow a geographical characterization of different EVOO samples. Principal component analysis (PCA) and hierarchical cluster analysis (HCA) (Gumus et al., 2017; Luka and Akun, 2019; Russo et al., 2020; Savio et al., 2014), linear discriminant analysis (LDA) (Benincasa et al., 2007; Cabrera-Vique et al., 2012; Beltran et al., 2015; Damak et al., 2019), classification trees (CTs) (Gumus et al., 2017), and artificial neural networks (ANNs) (Farmaki et al., 2012; Gonzalez-Fernandez et al., 2019) have been used most.

Several beneficial implications of EVOO are derived from its antioxidant content (Dugo et al., 2020; Hannachi and Elfalleh, 2020). Intake of antioxidant compounds from oil, such as phenols, phenolic acids, and flavonoids (Capriotti et al., 2014), is usually related to health well-being. As well known, natural antioxidants play a key role in contrasting reactive species activity in living organisms, thus preventing oxidative stress-related diseases, such as cardiovascular and neurodegenerative illness and many other chronic disorders (Pérez-Jiménez et al., 2008; Cioffi et al., 2010; Šarolić et al., 2014). Moreover, antioxidants prevent lipid oxidations that cause quality degradation and unpleasant taste formation in edible oils (Christodouleas et al., 2015). Therefore, estimation of antioxidant capacity is crucial for evaluating oil’s healthy and organoleptic properties. One of the most widely used *in vitro* procedures to routinely and globally estimate oil antioxidant power is the 2,2-diphenyl-1-picrylhydrazyl spectrophotometric assay (DPPH) that has the possibility of being easily applied to a high number of samples, allowing a great level of reliability (Kedare and Singh, 2011; Frezzini et al., 2019). The assay is based on the quantitative measurement of the decrease of absorbance due to the scavenging capacity of antioxidants present in the sample toward DPPH free radicals (Christodouleas et al., 2015).

All the described aspects making trace element determination, as well as the antioxidant activity of EVOO samples, are very important for both economic and health contexts (Zaroual et al., 2021; Bajoub et al., 2018). In particular, the European Union is the first producer, consumer, and exporter of olive oil in the world (Eurostat, 2019; IOC, 2018a,b). Italy follows Spain, the first world producer with an average of 20% of the total European olive oil production. About two-thirds of total Italian production is represented by EVOO (Carbone et al., 2018). Therefore, the use of a rapid and accurate analytical method for trace element analysis in EVOO has a great importance in quality control and food analysis (Llorent-Martínez et al., 2011; Shah and Soylak, 2021). Unfortunately, the determination of trace elements in EVOO samples is particularly difficult to perform, as some of them are present at very low concentrations and due to high complexity of the matrix (Shah and Soylak, 2021; Trindade et al., 2015). Sample preparation of EVOO samples is a critical step, and the determination of trace elements in EVOO requires very sensitive instrumental techniques such as inductively coupled plasma–mass spectrometry (ICP-MS) (Astolfi et al., 2021b).

The main purpose of this study is to evaluate the most significant relationships between element levels in EVOO and different categorizations, mostly related to the geographical origin using chemometric tools coupled with the ICP-MS method. For this purpose, 45 elements from a total of 237 EVOO samples from 15 Italian regions were analyzed. Also, the antioxidant activity of oils’ hydrophilic fraction (HF) was estimated by the DPPH assay. The corresponding data set constituted the basis for building and validating classification models for the discrimination of the samples according to specific categorizations, which reflect possible quality attributes of the oils (and for which there could be a statistically significant number of individuals available). In particular, discriminant classification models were built using partial least square discriminant analysis (PLS-DA) to account for the possibility of dealing with correlated variables and low samples to variable ratios; moreover, to evaluate model stability and, at the same time, their reliability in an unbiased way, also in cases where the available number of samples per category was not too large, a repeated double cross-validation strategy (rDCV) was adopted.

## 2 Materials and methods

### Sample collection

EVOO samples (N = 237) were collected between 2017 and 2018 from 15 production regions of Italy and different cultivars. In particular, a total of 64 EVOO samples were with PDOs and 21 with PGIs (European Union (EU), 2012). Table 1 summarizes the number of EVOO samples according to their geographical provenances in terms of the regions. All samples (∼100 mg) were kept in screw-capped glass vials in the dark at room temperature until analysis.

**TABLE 1:** Number of samples of extra virgin olive oil for each considered category.

	Region	All samples	Oil production			Cultivar (number of samples)
			Organically	Non-organically	Not reported	
Northern Italy	Trentino Alto Adige	7	2	4	1	Blend (3); Casaliva (1); Coratina (2)
	Liguria	6	0	4	2	Lavagnina (1); Taggiasca (4)
	Lombardy	3	0	2	1	Blend (1); Casaliva (1); Leccino (1)
	Veneto	3	1	2	0	Blend (2); Grignano (1)
	Emilia Romagna	1	0	1	0	Careggiolo (1)
Central Italy	Abruzzo	14	3	11	0	Blend (8); Dritta (3); Intosso (3)
	Lazio	24	6	8	10	Blend (5); Canino (3); Frantoio (1); Itrana (2); Leccino (2); Rosciola (1)
	Marche	7	2	5	0	Ascolana (2); Blend (1); Leccino (1); Orbetana (1); Raggiola (2)
	Toscany	79	33	42	4	Blend (38); Arancino (1); Coratina (1); Frantoio (10); Leccino (4); Moraiolo (7); Nocellara (1); Olivastra Seggianese (1); Pendolino (1); Raggiolo (1)
	Umbria	8	0	8	0	Blend (8)
Southern Italy	Apulia	33	6	18	9	Blend (3); Coratina (18); Frantoio (1); Leccino (1); Ogliarola (2); Olivastra (1); Peranzana (4); Pichioline (2)
	Calabria	12	5	6	1	Blend (3); Carolea (2); Nocellara (1); Ottobratica (4)
	Campania	7	0	7	0	Blend (2); Cammarotana (1); Ortice (1); Ravece (1); Salella (1)
	Sardinia	12	1	11	0	Bosana (3); Blend (6); Semidana (1)
	Sicily	21	4	14	3	Blend (4); Biancolilla (2); Cerasuola (1); Leccio del Corno (1); Nocellara (9); Tonda Iblea (3)

### Chemicals

All the solutions were prepared with deionized water (18.3 MΩ cm resistivity) obtained from an Arioso (Human Corporation, Seoul, Korea) Power I RO-UP Scholar UV deionizer system. HNO_3_ at 67% (suprapure; Carlo Erba Reagents, Milan, Italy), H_2_O_2_ at 30% (suprapure; Merck KgaA, Darmstadt, Germany), and Ar, He, and H_2_ gases at 99.9995% (SOL Spa, Monza, Italy) were used.

For ICP-MS analysis, all calibration standard solutions were prepared from a 1,000-mg l^−1^ multielement standard solution (VWR International, Milan, Italy) by dilution with 10% (v/v) HNO_3_ and H_2_O_2_ (2:1 v/v). Single standard solutions of In, Rh, Sc, and Th (at 0.010 mg l^−1^ from 1,000 ± 5 mg l^−1^; Merck KGaA, Darmstadt, Germany) and Y (at 0.005 from 1,000 ± 2 mg l^−1^; Panreac Química, Barcelona, Spain) were used as internal standards. A multielemental solution containing Ba, Be, Ce, Co, In, Pb, Mg, Tl, and Th (at 0.005 mg l^−1^ from 10.00 ± 0.05 mg l^−1^; Spectro Pure, Ricca Chemical Company, Arlington, TX, USA) was used to check the instrument performance.

For the estimation of the antioxidant activity of EVOO samples, DPPH was purchased from Sigma Aldrich Co. (St. Louis, MO, USA).

### Sample preparation and analysis

#### 2.1.1 Analysis of elements

Duplicate samples (∼0.5 g) of each EVOO variety were accurately weighed in 10-ml disposable graduated tubes (Artiglass, Due Carrare, PD, Italy). Then, 5 ml reagent mixture of 10% (v/v) HNO_3_ and H_2_O_2_ (2:1 v/v) was added to each tube and heated in a water bath (WB12, Argo Lab, Modena, Italy) at 95°C for 40 min (Astolfi et al., 2021b). The lower aqueous phase was transferred into a clean tube and subjected to the ICP-MS (820-MS; Bruker, Bremen, Germany) analysis without further dilutions. The elements were monitored in standard and collision–reaction interface (CRI) modes to check and reduce possible polyatomic interference, and the following isotopes were used: ^7^Li, ^9^Be, ^11^B, ^23^Na, ^24^Mg, ^27^Al, ^28^Si, ^31^P, ^39^K, ^44^Ca, ^49^Ti, ^51^V, ^52^Cr, ^55^Mn, ^57^Fe, ^59^Co, ^60^Ni, ^65^Cu, ^66^Zn, ^71^Ga, ^75^As, ^78^Se, ^85^Rb, ^88^Sr, ^90^Zr, ^93^Nb, ^98^Mo, ^107^Ag, ^112^Cd, ^118^Sn, ^121^Sb, ^125^Te, ^133^Cs, ^137^Ba, ^139^La, ^140^Ce, ^141^Pr, ^146^Nd, ^159^Tb, ^163^Dy, ^182^W, ^205^Tl, ^208^Pb, ^209^Bi, and ^238^U. CRI was used with He (30 ml min^−1^) and H_2_ (70 ml min^−1^) as cell gases. The ICP-MS operating conditions and parameters were as follows: radiofrequency power 1,400 W; plasma Ar flow rate 18 l min^−1^; auxiliary Ar flow rate 1.8 l min^−1^; nebulizer gas flow rate 0.9 l min^−1^; peak hopping scanning mode; steady-state analysis mode; dwell time between 50 and 100 ms, pump rate 3 rpm; five scans/replicate; and three replicates/sample. For the quantitative analysis of EVOO samples, calibration curves were built on seven different concentrations between 0.00025 and 0.05 mg l^−1^ and 0.0125 and 5 mg l^−1^ for all trace and major elements, respectively.

#### 2.1.2 Estimation of antioxidant activity

DPPH assay was performed according to the procedure described by Šarolić et al. (2014) with slight modifications. In detail, ∼0.5 g of each EVOO sample was mixed with 1 ml of 80:20 (v/v) CH_3_OH:H_2_O, and the mixture was blended in an ultrasonic bath (PROCLEAN 10.0 ultrasonic cleaner; Ulsonix, Berlin, Germany) for 15 min at 30°C. When the two phases appeared, the hydrophilic phase was collected, and the extraction was repeated another two times. Then, the hydrophilic extracts were combined to get a homogeneous sample. To perform DPPH assay, 50 µl of the HF sample was added to 2 ml of methanolic DPPH (0.04 mM), then the mixture was shaken for 30 min by rotating agitation (60 rpm; rotator; Glas-Col, Terre Haute, IN, USA) at room temperature in the dark and analyzed by UV-Vis spectrophotometry (Varian Cary 50 Bio UV-Vis; Varian Inc., Palo Alto, CA, USA) set at 517 nm, by measuring the sample absorbance decrease against the control (blank solution). Solutions were prepared daily and used fresh, and three replicates of each type of oil were performed. The DPPH radical scavenging activity was calculated in terms of percentage reduction of DPPH according to the following equation:

$$\text{DPPH }\left[\text{\%}\right]=\;\frac{\left({\text{A}}_{0}-{\text{A}}_{\text{S}}\right)}{{\text{A}}_{0}}\times 100$$

where A_0_ represents the absorbance of the blank solution and A_S_ is the absorbance of the sample.

### Quality assurance

The method accuracy for element determination was checked by recovery assays in the EVOO samples adding element at the low (0.005 and 0.02 mg l^−1^) and high (0.2 and 1 mg l^−1^) spike concentrations for all trace and major elements (B, Ca, K, Mg, Na, P, Si, and Sr) and always in the linear calibration range. In addition, accuracy was tested by a certified reference material (Conostan S-21; lot number: 21550100) obtained from SCP SCIENCE (Baie D’Urfé, Canada). The recoveries fell within 20% of the expected value and reproducibility lower than 20% (Astolfi et al., 2021b). The method detection and quantification limits (MDL and MQL, respectively) were in the range 0.004–510 and 2.5–5,000 μg kg^−1^, respectively. Only the Ca, Cr, Mg, Mn, Ni, P, Rb, Ti, and Zn levels in the EVOO samples were 100% greater than the MDL. The possible instrumental drift for the ICP-MS analysis was checked and corrected using an internal standard solution of In, Rh, Sc, Th, and Y (Astolfi et al., 2021b; 2020c). Blank samples and control standards were tested every 20 samples in each run, and recalibration was performed every 100 samples.

### Statistical analysis

The data were statistically evaluated according to the procedures of the software SPSS Statistics 25 (IBM Corp., Armonk, NY, USA) for univariate analysis. Analytical replicates were averaged prior to the successive elaboration. Non-parametric tests (Kruskal–Wallis and pairwise *post-hoc*) were applied because of the unequal numbers of samples per group and the not normal distribution (Soliani, 2003). The element concentrations measured below MDL were substituted by its half value (MDL/2) for the statistical elaboration (Farmaki et al., 2012). A *p*-value lower than 0.05 was considered statistically significant.

Partial least square discriminant analysis (PLS-DA; Ståhle and Wold, 1987; Barker and Rayens, 2003) implemented through in-house written functions running under the Matlab environment (R2015b, v.8.6, The MathWorks Inc., Natick, MA, USA) was used to build multivariate classification models. PLS-DA is a regression-based classification model which operates by coding class belonging by means of a dummy binary response matrix (or vector, when the problems involve only pairs of classes, as in the present study). In particular, if discrimination is sought between two categories, class belonging of the training samples is described by the vector y, having 1 in correspondence of all the individuals from the first class and 0 in all the remaining positions (i.e., those corresponding to the second group). A PLS model (Wold et al., 1983) is then built between the experimental data X and the dummy vector y, and the predicted value of the response ( $\hat{y})$ constitutes the basis for the classification of the samples: since the predicted responses are real-valued, an optimal threshold $y_{thres}\;$ has to be calculated so that, if the predicted response is greater than $y_{thres}$ , the sample is predicted as class 1, otherwise as class 2. In the present study, the threshold was calculated by applying LDA on the predicted responses calculated on the training samples (Perez et al., 2009).

The reliability of the classification models was evaluated by means of a repeated double-cross-validation (rDCV) procedure (Filzmoser et al., 2009). Double cross-validation (DCV) is a validation strategy which involves two nested loops of cross-validation: an inner loop for model selection (i.e., for choosing the optimal number of latent variables) and an outer loop which mimics an external (i.e., not involved in any model building and/or optimization stage) test set, to be used for estimating the prediction and generalization ability. In order to avoid that the performances of the model depend on a particular sample splitting scheme, the procedure is repeated a sufficient number of times, changing the distribution of the individuals across the different cancelation groups, hence the name “repeated” DCV. Repeating the double-cross-validation procedure allows also having multiple predictions for the same samples, which translates to the possibility of estimating confidence intervals for all the classification figures of merit and model parameters.

## 3 Results and discussion

### Levels of elements

Table 2 shows the concentration of the elements in EVOO from all over Italy. The content of Tl was below the respective MDL (0.06 µg kg^−1^) in all the samples. Si, Te, and Nb were found above the MDL (270, 0.03, and 0.04 µg kg^−1^, respectively) only in 1%, 3%, and 7% of all samples. Only the Ca, Cr, Mg, Mn, Ni, P, Ti, and Zn levels in the EVOO samples were 100% greater than the MDL. The maximum concentrations for As (4.0 μg kg^−1^), Cu (41.6 μg kg^−1^), Fe (582 μg kg^−1^), and Pb (22.1 μg kg^−1^) were lower than the MRLs established by the IOC for olive and pomace-olive oils, which are 100 μg kg^−1^ for As, Cu, and Pb and 3,000 μg kg^−1^ for Fe (International Olive Council, 2009). Calcium showed the highest concentration ranging from 1,230 to 35,700 μg kg^−1^, whereas from 10- to 50-fold lower levels were found for Fe, Mg, Na, P, and Zn (median = 77, 91, 110, 272, and 111 μg kg^−1^, respectively).

**TABLE 2:** Method detection limits (MDL; μg kg^−1^) and element levels [median, minimum (min) and maximum (max); μg kg^−1^] in extra-virgin olive oils (EVOO; *n* = 237) from all over Italy.

Element	MDL	Italian EVOO samples			
		%N > MDL	Median	Min	Max
Ag	0.06	27	<0.06	<0.06	0.86
Al	9	85	34	<9	1,300
As	0.3	28	<0.3	<0.3	4.0
B	20	16	<20	<20	770
Ba	0.7	49	<0.7	<0.7	175
Be	0.004	43	<0.004	<0.004	0.431
Bi	0.1	23	<0.1	<0.1	1.0
Ca	510	100	4,090	1,230	35,700
Cd	0.07	67	0.09	<0.07	0.97
Ce	0.1	65	0.2	0.1	3.5
Co	0.05	70	0.12	<0.05	2.16
Cr	0.3	100	5	0.4	839
Cs	0.007	55	0.008	<0.007	0.101
Cu	0.6	99	3.2	<0.6	41.6
Dy	0.005	19	<0.005	<0.005	0.055
Fe	12	99	77	<12	582
Ga	0.06	15	<0.06	<0.06	0.69
K	40	24	<40	<40	939
La	0.05	70	0.10	<0.05	0.79
Li	0.06	32	<0.06	<0.06	6.07
Mg	10	100	91	21	723
Mn	0.5	100	2.4	1.1	43.5
Mo	0.3	20	<0.3	<0.3	2.0
Na	25	98	110	<25	585
Nb	0.04	7	<0.04	<0.04	0.11
Nd	0.03	52	0.03	<0.03	13.8
Ni	0.5	100	5.6	2.1	49.7
P	60	100	272	127	650
Pb	0.3	99	0.9	<0.3	22.1
Pr	0.008	40	<0.008	<0.008	1.65
Rb	0.06	99	0.24	<0.06	1.77
Sb	0.02	16	<0.02	<0.02	0.37
Se	0.6	48	<0.6	<0.6	7.8
Si	270	1	<270	<270	3,340
Sn	0.06	63	0.08	<0.06	1.94
Sr	1	65	3	1	58
Tb	0.006	19	<0.006	<0.006	1.28
Te	0.03	3	<0.03	<0.03	0.06
Ti	0.4	100	1.9	0.8	10.7
Tl	0.06	0	<0.06	<0.06	<0.06
U	0.005	30	<0.005	<0.005	0.050
V	0.08	98	0.53	<0.08	1.40
W	0.3	38	<0.3	<0.3	5.1
Zn	20	100	111	54	749
Zr	0.1	57	<0.1	<0.1	2.3

Concentrations of elements obtained in this study were compared to levels measured in EVOO from several other Mediterranean countries (Supplementary Tables S1–S4). Levels of many elements showed wide variability even within the same country. The Ag, Ba, P, and Sn data were not considered because these elements are not completely extracted with the method used. As regards the content of B, Be, Dy, Nd, Pr, Si, Tb, and Te, we could not find other data for EVOO in the literature. Our results were similar to those reported by another study on Italian EVOO (Benincasa et al., 2007); on the contrary, they differed significantly from other data concerning most of the elements investigated in the EVOOs of Spain (Beltran et al., 2015; Llorent-Martínez et al., 2014), Croatia (Pošćić et al., 2019), Tunisia (Damak et al., 2019), and Turkey (Gumus et al., 2017). The concentrations of Ca (1,230–35,700 μg kg^−1^), Cr (0.4–839 μg kg^−1^), Mg (21–723 μg kg^−1^), and Ni (2.1–49.7 μg kg^−1^) found in this study were in the same range to that found in other Italian EVOO (Ca = 1850–26,900 μg kg^−1^; Cr = 116–437 μg kg^−1^; Mg = 56–1,030 μg kg^−1^; and Ni = nd-46.9 μg kg^−1^) as reported by Benincasa et al. (2007), but from 10 to 100 times higher than the levels reported in Croatian (Pošćić et al., 2019) and Turkish EVOO (Gumus et al., 2017). Fe concentrations (<12–582 μg kg^−1^) varied from 100 times lower to 100 times higher than the level of Fe quantified in EVOO from Turkey (1–14,670 μg kg^−1^) by Gumus et al. (2017) and Croatia (0.19–2.57 μg kg^−1^) by Pošćić et al. (2019) or Spain (0.5–1.2 μg kg^−1^) by Beltran et al. (2015), respectively. This variability in the concentrations of the elements present in EVOO samples may depend on various factors related to the geochemistry of the provenance soil but also to physiological aspects typical of the species from which a particular EVOO derives (Giaccio and Vicentini, 2008).

Grouping the data according to geographic origin as north (Emilia Romagna, Liguria, Lombardy, Trentino Alto Adige, and Veneto), center (Abruzzo, Lazio, Marche, Tuscany and Umbria), and south (Apulia, Calabria, Campania, Sardinia and Sicily) of Italy, it is possible to identify elements that differ significantly from one group to another (Table 3). In particular, the EVOO samples from northern Italy had significantly higher levels of Cs, Fe, Na, P, and Pr than those from central Italy and Fe, Pr, and U than those from southern Italy. Both Fe and Pr appear to provide a good tool for tracing the EVOO production chain in accord with other authors (Aceto et al., 2019; Damak et al., 2019). Iron is common in silicates and carbonates present in soil (Pohl, 2011); however, some authors reported that Fe may be present in edible oils as a result of storage and processing contaminations (Mendil et al., 2009; Zeiner et al., 2010). Praseodymium and the other lanthanides do not have a defined role in the metabolism of plants; therefore, their distribution remains almost unchanged in the passage from the soil to the fruits (Aceto et al., 2019). For this reason, these elements can be used as fingerprints to discriminate the geographic origin of the EVOO samples (Farmaki et al., 2012; Aceto et al., 2019). In addition, the analysis of some elements in EVOO, such as Cs and Rb, which can be easily mobilized in the soil, can be linked to a geogenic source rather than an anthropogenic origin (such as extraction process or cultivation practices) and can help in the geographical traceability of EVOO samples (Kelly, Heaton, & Hoogewerff, 2005).

**TABLE 3:** Element levels [median, minimum (min) and maximum (max); μg kg^−1^] in extra-virgin olive oils from north (n = 20), central (*n* = 132) and south (*n* = 85) Italy.

Element	North Italy^a^				Central Italy^b^				South Italy^c^			
	%N > MDL	Median	Min	Max	%N > MDL	Median	Min	Max	%N > MDL	Median	Min	Max
Ag	25	<0.06	<0.06	0.26	30	<0.06	<0.06	0.86	25	<0.06	<0.06	0.22
Al	75	32	<9	615	86	34	<9	1,291	86	34	<9	1,298
As	25	<0.3	<0.3	2.8	28	<0.3	<0.3	4.0	28	<0.3	<0.3	2.2
B	20	<20	<20	85	15	<20	<20	734	16	<20	<20	770
Ba	50	0.6	0.4	99.5	49	2.9	<0.7	175	48	<0.7	<0.7	147
Be	40	<0.004	<0.004	0.431	37	<0.004	<0.004	0.272	49	0.004	<0.004	0.061
Bi	40	<0.1	<0.1	0.2	23	<0.1	<0.1	0.4	19	<0.1	<0.1	1.0
Ca	100	4,590	1,480	9,170	100	3,648	1,229	35,709	99	4,278	1,432	24,122
Cd	70	0.12	<0.07	0.33	66	0.09	<0.07	0.97	67	0.09	<0.07	0.61
Ce	70	0.2	<0.1	0.7	69	0.2	0.1	3.5	60	0.2	0.1	1.0
Co	90	0.11	<0.05	0.59	68	0.13	<0.05	1.23	69	0.08	<0.05	2.16
Cr	100	3.7	0.5	839	99	5.0	0.4	123	100	4.1	0.5	533
Cs	80	0.013^a^	<0.007	0.080	52	0.007^a^	<0.007	0.084	55	0.008	<0.007	0.101
Cu	100	4.6	<0.6	20.7	99	3.0	<0.6	40.9	100	3.3	<0.6	41.6
Dy	20	<0.005	<0.005	0.010	20	<0.005	<0.005	0.026	17	<0.005	<0.005	0.055
Fe	100	158^a^ ^,^ ^b^	<12	495	99	70^a^	<12	403	100	86^b^	14	582
Ga	35	<0.06	<0.06	0.33	11	<0.06	<0.06	0.69	16	<0.06	<0.06	0.59
K	40	<40	<40	293	20	<40	<40	673	26	<40	<40	939
La	80	0.13	<0.05	0.41	70	0.08	<0.05	0.79	67	0.11	<0.05	0.71
Li	40	<0.06	<0.06	1.67	29	<0.06	<0.06	6.07	35	<0.06	<0.06	4.42
Mg	100	97	37	262	100	90	21	723	99	96	28	613
Mn	100	2.7	1.5	7.1	99	2.3	1.1	18.6	100	2.6	1.4	43.5
Mo	30	<0.3	<0.3	1.3	19	<0.3	<0.3	1.7	20	<0.3	<0.3	2.0
Na	100	131^a^	87	331	99	102^a^	<25	585	100	114	<25	513
Nb	15	<0.04	<0.04	0.05	5	<0.04	<0.04	0.06	8	<0.04	<0.04	0.11
Nd	80	0.06	<0.03	1.39	47	<0.03	<0.03	6.43	53	0.03	<0.03	13.8
Ni	100	5.2	2.5	29.5	100	6.0	2.1	40.6	100	5.4	2.4	49.7
P	100	309^a^	220	650	99	269^a^	127	522	100	272	189	548
Pb	100	1.2	<0.3	4.3	100	0.8	<0.3	22.1	100	1.1	<0.3	8.7
Pr	70	0.012^a^ ^,^ ^b^	<0.008	0.359	38	**<**0.008^a^	<0.008	1.58	35	**<**0.008^b^	<0.008	1.65
Rb	95	0.29	<0.06	1.10	100	0.24	0.06	1.77	100	0.26	<0.06	1.36
Sb	10	<0.02	<0.02	0.04	17	<0.02	<0.02	0.37	16	<0.02	<0.02	0.14
Se	55	0.6	<0.6	6.8	49	<0.6	<0.6	6.9	44	0.6	<0.6	7.8
Si	0	<270	<270	<270	1	<270	<270	3,344	1	<270	<270	442
Sn	80	0.10	<0.06	0.45	59	0.06	<0.06	0.60	65	0.09	<0.06	1.94
Sr	80	3	1	7	64	3	1	34	63	3	1	58
Tb	25	<0.006	<0.006	0.112	20	<0.006	<0.006	1.13	16	<0.006	<0.006	1.28
Te	5	<0.03	<0.03	0.05	1	<0.03	<0.03	0.05	6	<0.03	<0.03	0.06
Ti	100	2.2	1.2	8.1	99	1.8	0.8	5.6	100	2.1	1.1	10.7
Tl	0	<0.06	<0.06	<0.06	1	<0.06	<0.06	0.08	0	<0.06	<0.06	0.03
U	55	0.006^a^	<0.005	0.044	31	<0.005	<0.005	0.044	24	**<**0.005^a^	<0.005	0.050
V	100	0.50	<0.08	1.04	99	0.52	<0.08	1.21	100	0.55	<0.08	1.40
W	40	<0.3	<0.3	2.0	37	<0.3	<0.3	2.3	39	<0.3	<0.3	5.1
Zn	100	145	55	283	99	98	54	749	99	143	57	672
Zr	55	0.1	0.1	0.6	53	0.1	0.1	1.8	63	0.1	0.1	2.3

a North Italy groups the following regions: Emilia Romagna, Liguria, Lombardy, Trentino Alto Adige, and Veneto.

b Central Italy groups the following regions: Abruzzo, Lazio, Marche, Tuscany, and Umbria.

c South Italy groups the following regions: Apulia, Calabria, Campania, Sardinia, and Sicily. For each element, numbers in bold with the same superscript indicate significant differences (*p* < 0.05).

By comparing the concentrations of the elements in the EVOO samples from each region (Supplementary Tables S5–S7), the number of elements that differ significantly increases. Table 4 shows a summary of all the elements that differ significantly according to the region. Emilia Romagna was not considered for the comparison because there was only one EVOO sample to consider. EVOOs from Lombardy did not have levels of elements that are significantly different from those of oils from all other regions. Considering the other oils of northern Italy, the EVOOs from Trentino and Liguria differed significantly from the EVOOs from Marche only for the content of Na, which in the EVOOs from Marche (median = 38 μg kg^−1^) was about four times lower, while the EVOOs from Veneto had a higher content of Fe (median = 218 μg kg^−1^) than the oils from Abruzzo (median = 15 μg kg^−1^) and a higher content of Fe and Na (median = 218 and 174 μg kg^−1^, respectively) compared to the Marche. The EVOO samples from Lazio differed significantly for a large number of elements (Ba, Ca, Cd, Ce, Cs, Dy, Ga, La, Mg, Na, Nd, Pr, Pb, Rb, Sb, Sr, Tb, Ti, U) compared to Tuscany, Abruzzo, Campania, and Marche. In all cases, levels of Cd (median = 0.14 μg kg^−1^), La (median = 0.20 μg kg^−1^), and Rb (median = 0.48 μg kg^−1^) were higher than those of oils from other regions mentioned above.

**TABLE 4:** Summary of significant differences within medians of the 45 selected elements and antioxidant activity (DPPH%) among all samples from Italian regions by Kruskall–Wallis and pairwise *post-hoc* tests. A *p*-value lower than 0.05 was considered statistically significant.

	Trentino	Liguria	Veneto	Lazio	Tuscany	Umbria	Calabria	Apulia	Sardinia	Sicily
Toscany		DPPH%		Cd, Cs, Dy, Ga, La, Na, Nd, Pr, Rb, Sb, Tb, Ti, U	-					
Umbria				Dy, U		-				
Apulia					Ti,Zr		-			
Sardinia				La, Tb, U				Al	-	
Sicily				U	Be,DPPH%			DPPH%	Be	-
Abruzzo		DPPH%	Fe	Ba, Ca, Cd, Ce, Dy, La, Mg, Nd, Rb, Sr, Tb	Ni	Fe,Se	Fe	Ce,La,Ni,Zn,Zr		As,Ba,Ca,Ce,Fe,LaNi, Zn, DPPH%
										
Campania				Ba, Cd, La, Mg, Na, Rb, U			Na	La	Na	Ba,La
Marche	Na	Na	Fe,Na	Ba, Cd, Ce, Cs, La, Na, Nd, Mg, Pb, Pr, Rb, Ti, U		Fe	Cd,Fe,Na	Na,Rb,Ti	Na	Ba,Be,Rb

### Antioxidant activity

Following the extraction and storage of EVOO, it is inevitable that an oxidation process occurs, which leads to a deterioration of the oil (Bendini et al., 2007). Some factors such as temperature, light, oxygen and other chemical elements, unsaturated fatty acid composition, and the presence of antioxidants can affect the oxidation process differently (Frankel 1985). Phenolic compounds have antioxidant capacities in EVOO since they can eliminate peroxyl and alkoxy radicals and chelate transition metal ions present in traces (Visioli et al., 1998). Several elements are known for their antioxidant properties (Perna et al., 2012; Thiruvengadam et al., 2020). Indeed, in the present study (Supplementary Table S8), significant correlations were observed between the antioxidant activity and elements. A positive and significant moderate correlation (r = 0.500–0.768, *p* = 0.05) was observed between Al, Ca, Fe, V, and Zr in EVOOs from Abruzzo, Ba in EVOOs from Apulia, and B, Mn, Se, and V in EVOOs from Sardinia and the DPPH% data. Conversely, a low and positive correlation (r < 0.4) was recorded between Ba and Ni and the antioxidant activity of all samples. Other elements (Ag, Cr, Cu, Li, Sb, Si, and Tl) might not affect the antioxidant properties as non-significant correlations were observed between them.

Supplementary Tables S5–S7 show the antioxidant activity measured by the DPPH assay (DPPH%) in the EVOO samples from each region. EVOOs from central and southern Italy showed higher antioxidant activity than oils from northern Italy. In particular, Table 4 shows that EVOOs from Sicily had a significantly lower DPPH% (median = 18.2%) than oils from Abruzzo (median = 47%), Apulia (median = 37.6%), and Tuscany (median = 36.2%), while the EVOOs from Liguria had significantly lower DPPH% (median = 15.7%) compared to Tuscany. The highest data of DPPH% (67.3%) was found in the oils of Campania. Cioffi et al. (2010) demonstrated that oils from Campania have antioxidant properties, which are very likely due to the presence of high contents of phenolic compounds.

### Classification of EVOOs according to geographical origin

At first, the possibility of discriminating the different EVOOs according to their geographical origin was considered. In particular, due to the unavailability of the information about the origin of all the samples and to the unbalancedness in the distribution of samples per class, when considering the oils of known origin, several two-class models (i.e., comparing two regions at a time) were built and validated. Here it must be further stressed that all the regions for which the available number of certified individuals was too low to be considered representative have not been included in the comparison.

In all cases, PLS-DA analysis was carried out on the matrix made up of the concentrations of the elements presenting at least 70% of the values above the limit of detection (so to avoid possible artifacts related to data imputation) and including also TEAC and DPPH. Models were built after autoscaling and validated by means of an rDCV procedure with 50 runs, 10 cancelation groups in the outer loop (the one mimicking the external test set) and 5 in the inner loop (the one used for model selection, i.e., definition of the optimal number of latent variables). The results obtained are summarized in Table 5, where the accuracy, the mean correct classification rate, and the sensitivities for the two compared classes are reported. Since two-class discriminant models were calculated, due to symmetry the sensitivity of a class (true positive rate) is the specificity (true negative rate) of the other category; this is why sensitivities only have been reported. Moreover, since the number of samples per class was, in some cases, highly unbalanced (Table 1), we have decided to report both classification accuracy (percentage of correctly classified samples irrespectively of the category over the total number of samples) and the mean correct classification rate, which is the average of the specificities of the two classes.

**TABLE 5:** PLS-DA discrimination between pairs of geographical origin. Figures of merit estimated on the outer loop of the rDCV procedure (expressed as mean ± standard deviation).

Class1	Class2	% accuracy	Mean % correct classification rate	% sensitivity (Class1)	% sensitivity (Class2)
Lazio	Abruzzo	76.2 ± 3.9	77.1 ± 4.2	73.1 ± 3.9	81.0 ± 7.7
Lazio	Sicily	79.4 ± 3.1	79.0 ± 2.9	81.9 ± 4.9	76.1 ± 2.8
Lazio	Apulia	68.8 ± 5.2	68.9 ± 5.2	64.6 ± 6.2	73.2 ± 7.6
Lazio	Tuscany	75.2 ± 1.8	69.2 ± 2.3	57.8 ± 4.2	80.6 ± 2.1
Lazio	Calabria	61.7 ± 5.1	54.4 ± 5.5	71.9 ± 5.9	36.9 ± 8.5
Abruzzo	Calabria	81.4 ± 6.1	81.0 ± 7.1	82.9 ± 4.3	79.1 ± 12.2
Abruzzo	Sicily	75.0 ± 4.3	75.5 ± 4.4	81.2 ± 7.6	69.7 ± 5.9
Abruzzo	Tuscany	58.2 ± 3.4	54.6 ± 5.6	49.3 ± 11.6	59.9 ± 4.0
Abruzzo	Apulia	54.3 ± 6.6	54.2 ± 6.8	53.6 ± 9.6	54.8 ± 7.5
Sicily	Tuscany	69.5 ± 2.7	65.8 ± 4.1	59.9 ± 7.6	71.8 ± 2.8
Sicily	Apulia	70.7 ± 4.3	70.2 ± 4.2	65.4 ± 4.9	74.9 ± 6.5
Tuscany	Apulia	64.6 ± 3.1	55.1 ± 4.9	72.5 ± 2.9	37.7 ± 9.3

As anticipated in the materials and methods section, the use of repeated double cross-validation allows obtaining not only a point estimate of the figures of merit on the validation (outer loop) samples but also their confidence intervals, so as to be able to evaluate the consistency of the results.

By looking at Table 5, it is evident how the different models result in different reliabilities, with some presenting rather low classification performances. On the other hand, there are some models which result in an overall accuracy higher than 75%, with a comparable mean correct classification rate (suggesting that the classification performances are not affected by the numerosity of the samples; Table 1). Additionally, the standard deviation of the figures of merit for these models is relatively low (corresponding roughly to one more sample being correctly or wrongly classified with respect to the reported averages), confirming the consistency of the obtained classification. Based on these considerations, only the best models will be discussed in detail in the remainder of this section, namely, Lazio vs. Abruzzo, Lazio vs. Sicily, Abruzzo vs. Calabria and Abruzzo vs. Sicily.

**FIGURE 1:**
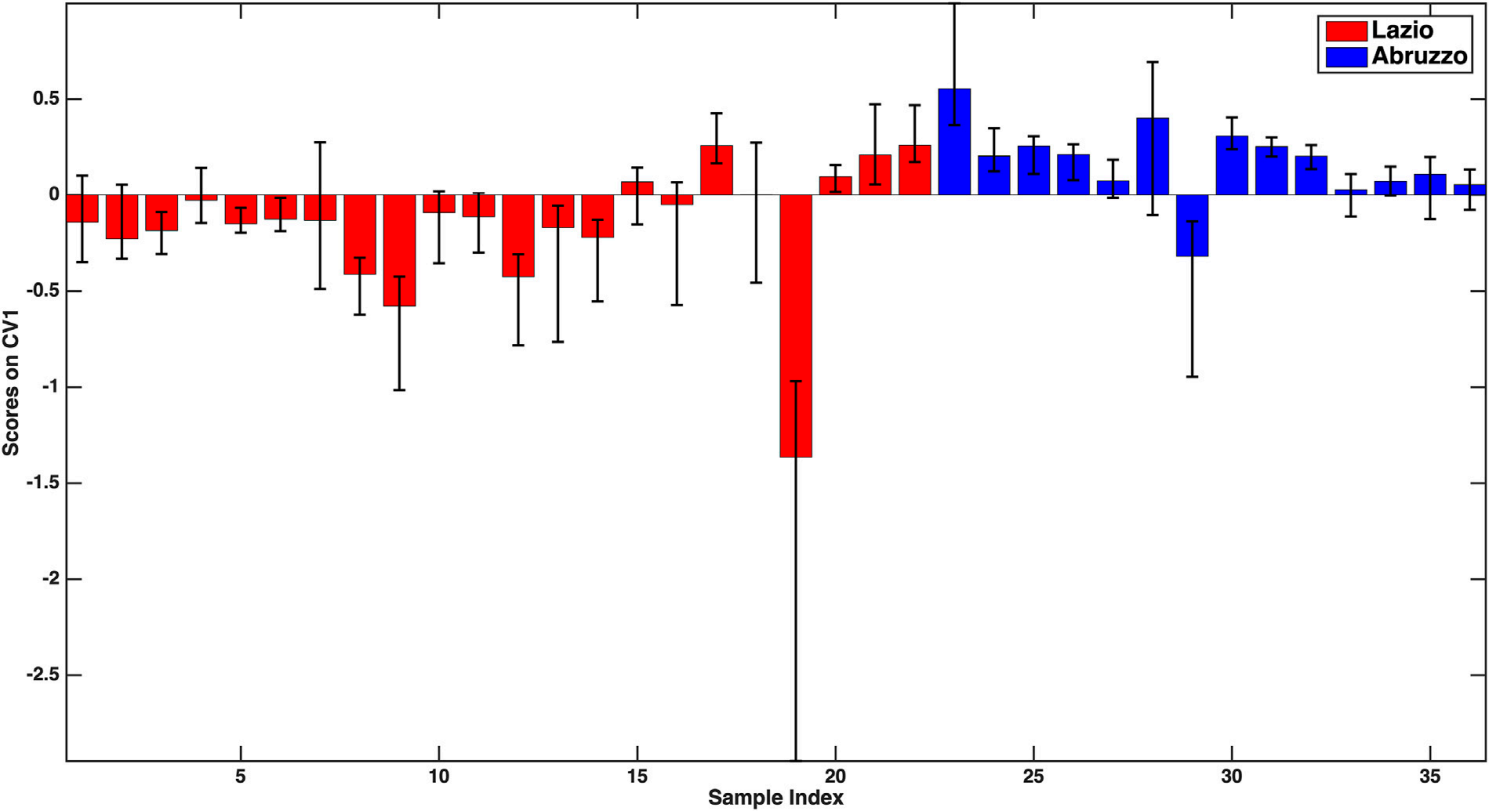
**–**PLS-DA model for the discrimination between Lazio and Abruzzo samples: mean scores of the rDCV outer loop samples along the only canonical variate of the model together with their 95% confidence intervals. Legend: red bars–Lazio; blue bars–Abruzzo.

The first model to be examined is the one discriminating Lazio samples from the oils from Abruzzo, for which an overall 76.2 ± 3.9% classification accuracy on the outer loop samples was registered. By looking at the individual sensitivities together with their confidence intervals (73.1 ± 3.9% for Lazio and 81.0 ± 7.7% for Abruzzo), it can be stated that the two categories are predicted comparably well. These results can also be graphically appreciated in Figure 1, where the mean scores of the outer loop samples along the only canonical variate of the model together with their 95% confidence intervals are displayed. It is evident from Figure 1 how almost all the Abruzzo samples have positive scores, while the large majority of Lazio samples are characterized by negative coordinates on the component, indicating a good separation between the categories.

For the sake of interpretation, another advantage of the rDCV procedure is that confidence intervals can also be calculated for model parameters, so as to be able to identify which are the variables that contribute significantly to the discrimination (e.g., by inspecting the values of the associated regression coefficients or of the VIP scores). Moreover, investigating the sign of the regression coefficients also allows postulating whether the associated predictor is more or less concentrated in a category with respect to the other. In particular, the variables found to significantly contribute to the discriminant model were V, Fe, Zn, Rb, antioxidant capacity (all higher in Lazio samples), and Ni and antioxidant activity in the DPPH assay (higher in the oils from Abruzzo).

**FIGURE 2:**
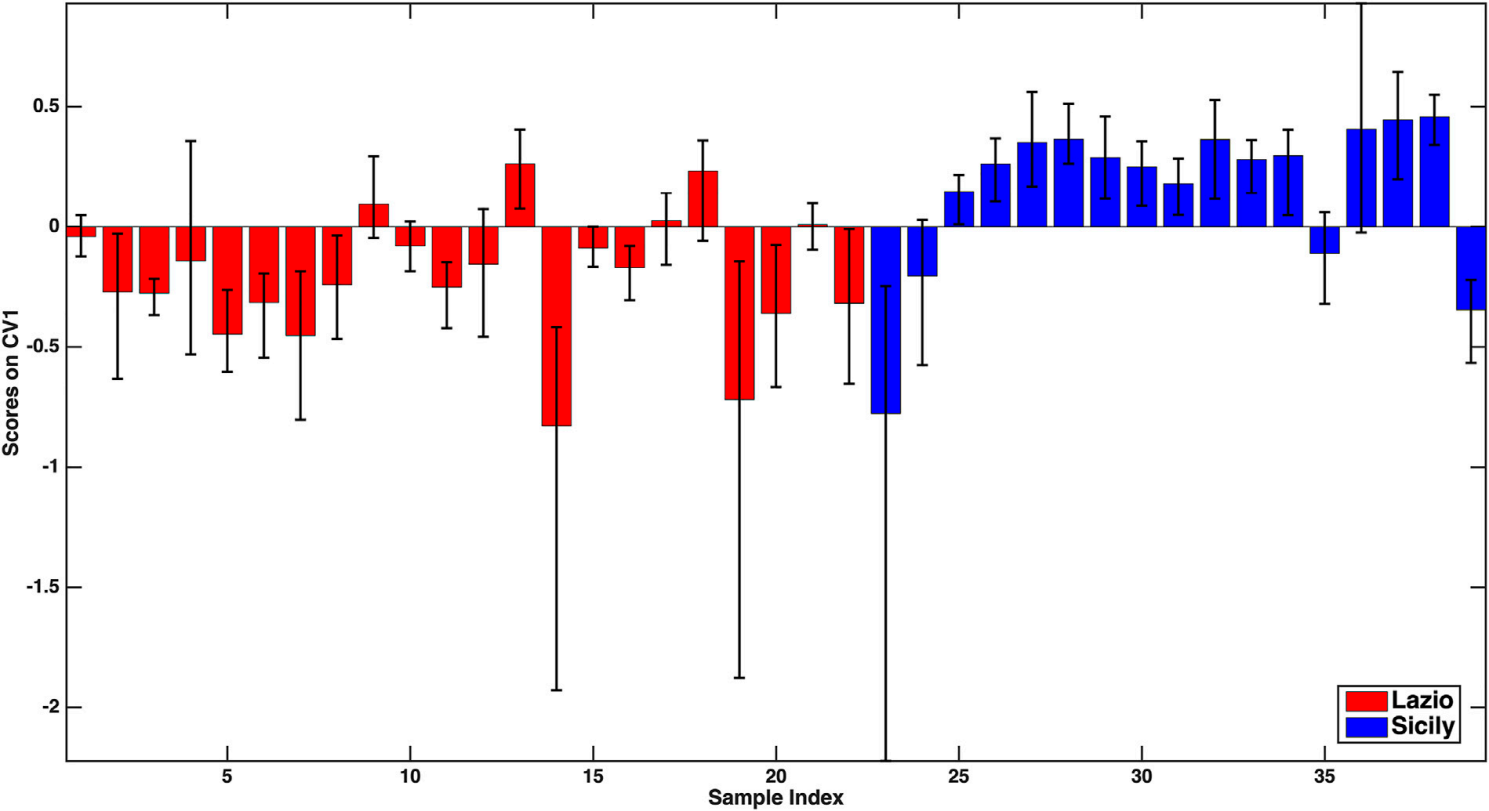
**–**PLS-DA model for the discrimination between Lazio and Sicily samples: mean scores of the rDCV outer loop samples along the only canonical variate of the model together with their 95% confidence intervals. Legend: red bars–Lazio; blue bars–Sicily.

As far as the Lazio vs. Sicily model is concerned, a slightly higher accuracy was obtained (79.4 ± 3.1%), the individual sensitivities being 81.9 ± 4.9% for Lazio and 76.1 ± 2.8% for Sicily. Analogously to that described above, the discrimination between the two classes can also be visually appreciated in Figure 2, where the mean scores of the outer loop samples along the only canonical variate of the model together with their 95% confidence intervals are displayed.

In this case, based on the values of the PLS-DA regression coefficients, all the variables found to significantly contribute to the discriminant model (Na, Mg, P, Ti, Rb, antioxidant activity in the DPPH assay) should be, on average, higher in the oils from Lazio. When considering the model discriminating Abruzzo oils from the Calabrian ones, an overall 81.4 ± 6.1% accuracy on the outer loop samples was obtained, the mean correct classification rate (81.0 ± 7.1%) and the individual sensitivities for the two categories (82.9 ± 4.35% for Abruzzo and 79.1 ± 12.2% for Calabria) being almost equal. In particular, the higher standard deviation of the sensitivity for Calabria is due to the very limited number of samples in that class. When looking at the significant predictors, only five variables (P, V, Fe, Zn, and antioxidant activity) were identified, and the coefficients indicate that they all should be, on average, higher for the Abruzzo samples.

Lastly, the Abruzzo vs. Sicily model resulted in an accuracy of 75.0 ± 4.3%, the sensitivities being 81.2 ± 7.6% for Abruzzo and 69.7 ± 5.9% for Sicily. Inspection of the model parameters led to identifying as significant the contribution of Na, Ni, and antioxidant activity (in the DPPH assay with higher data in the oils from Abruzzo) and of V and Fe, more concentrated in the samples from Sicily.

Classification of EVOOs according to cultivar and to whether it was organically produced.

In a second stage of the study, the possibility of discriminating oil samples according to their cultivar was explored. In this case, given the available information about the samples and the fact that only a relatively small fraction of the analyzed oils was monovarietal, the investigation was restricted to the comparison of Coratina (21 samples) and Frantoio (12 samples) (Table 1). The PLS-DA classification approach was validated through an rDCV strategy as described in the previous section and resulted in an overall accuracy of 68.9 ± 6.2%, and 80.7 ± 8.8% and 61.9 ± 7.6% sensitivities for Frantoio and Coratina, respectively, corresponding to a mean correct classification rate of 71.3 ± 6.2%. Investigation of the model parameters suggested that five variables only, namely, P, Ti, Zn (higher in Coratina), Fe, and Ni (more concentrated in Frantoio), significantly contributed to the discriminant model.

Lastly, the possibility of discriminating whether the oil was organically produced or not was also attempted, but the classification model resulted in a very poor accuracy (close to 50%) suggesting that, at least for the investigated samples, organic cultivation has little impact on the elemental composition with respect to non-organic production.

## 4 Conclusion

This study showed that the As, Cu, Fe, and Pb levels in the analyzed samples were far below the MRLs, which certifies the high quality of Italian EVOO.

The element concentrations allow to distinguish well some geographical origins of the EVOO samples and also, although slightly less well, the two cultivars Coratina and Frantoio. On the other hand, given the high heterogeneity of the data set, it is not possible to distinguish organic oils from non-organic ones. This is probably due to the fact that within the two classes the variability related to geographical origin and cultivar is added.

This study can be used to create datasets for element levels in EVOOs for each production region to support geographic origin authentication. In the future, other information will have to be considered together with the elemental profile of EVOO such as climatic factors and bioavailable fraction of the total content of elements to further corroborate the use of the elements as a marker of provenance.

## Data Availability

The original contributions presented in the study are included in the article/Supplementary Material; further inquiries can be directed to the corresponding author.
