# Supplementary material for: Multielement Characterization and Antioxidant Activity of Italian Extra-Virgin Olive Oils
Source: Front Chem. 2021 Nov 16;9:769620. doi: 10.3389/fchem.2021.769620 (PMC8635196; doi:10.3389/fchem.2021.769620)
Supplement: Supplementary file 1 [file Table1.docx]

Supplementary Material

Multi-element characterization and antioxidant activity of Italian extra-virgin olive oils

Maria Luisa Astolfi^1*^, Federico Marini^1^, Maria Agostina Frezzini^2^, Lorenzo Massimi^2^, Anna Laura Capriotti^1^, Carmela Maria Montone^1^, Silvia Canepari^2^

^1^Department of Chemistry, Sapienza University of Rome, Rome, Italy

^2^Department of Environmental Biology, Sapienza University of Rome, Rome, Italy

*** Correspondence:**Dr. Maria Luisa Astolfi, Department of Chemistry, Sapienza University of Rome, Rome, Italy.
[marialuisa.astolfi@uniroma1.it](mailto:marialuisa.astolfi@uniroma1.it)

**Table S1.** Element levels [mean or median (range minimum-maximum); μg kg^-1^] in extra-virgin or virgin olive oils from different Mediterranean countries.

| **Reference** | **Country** | **Al** | **As** | **B** | **Be** | **Bi** | **Ca** | **Cd** | **Ce** | **Co** | **Cr** |
| --- | --- | --- | --- | --- | --- | --- | --- | --- | --- | --- | --- |
| **This study** | Italy | 34 (<9-1300) | <0.3 (<0.3-4.0) | <20 (<20-770) | <0.004 (<0.004-0.431) | <0.1 (<0.1-1 | 4090 (1230-35700) | 0.09 (<0.07-0.97) | 0.2 (0.1-3.5) | 0.12 (<0.05-2.16) | 5 (0.4-839) |
| Benincasa et al., 2007 | Italy (Calabria, Apulia, Umbria and  Abruzzo) | - | 1.25-26.6 | - | nd-0.182 | - | 1850-26900 | 0.088-0.366 | - | 0.023-0.413 | 116-437 |
| Camin et al., 2010 | Italy (South: Calabria, Apulia and Sicily; Centre: Lazio, Tuscany and Umbria; North: Veneto and Trentino) | - | - | - | - | - | 395-950 | - | 0.002-4.72 | 0.003-0.031 | - |
| Russo et al., 2020 | Italy  (Campania region) | 2.68-9.60 | - | - | - | - | 46.7-143.3 | - | - | - | - |
| Pošćić et al., 2019 | Croatia | - | - | - | - | - | 27.8 (7.14–212) | 0.001 (0.001–0.002) | 0.002 (0.001–0.04) | 0.003 (0.001–0.01) | 0.005 (0.005–0.58) |
| Beltran et al., 2015 | Spain (four  Spanish municipalities of Huelva) | 0.3-1.1 | 5.3-7.7 | - | - | - | 5.1-11.2 |  |  | 7.9-11.0 | 51.9-123.3 |
| Cabrera-Vique et al., 2012 | Spain (pooled data)  Granada province  Jaén province | - | - | - | - | - | - | - | - | - | 6.84-99.2 |
| Llorent-Martínez et al., 2011 | Spain | - | <3 | - | - | - | - | - | - | - | - |
| Llorent-Martínez et al., 2014 | Spain | 150-250 | 50 | - | - | - |  | 150 | - | 110 | 250 |
| Damak et al., 2019 | Tunisia (four geographical  Origins) | - | 10-29 | - | - | - | - | - | - | - | - |
| Gumus et al., 2017 | Turkey | - | 0.00-0.060 | - | - | - | 130-730 | - | - | 0.000-0.065 | 0.00-1.47 |

**Table S2.** Element levels [mean or median (range minimum-maximum); μg kg^-1^] in extra-virgin or virgin olive oils from different Mediterranean countries.

| **Reference** | **Country** | **Cs** | **Cu** | **Dy** | **Fe** | **Ga** | **K** | **La** | **Li** | **Mg** | **Mn** |
| --- | --- | --- | --- | --- | --- | --- | --- | --- | --- | --- | --- |
| **This study** | Italy | 0.008 (<0.007-0.101) | 3.2 (<0.6-41.6) | <0.005 (<0.005-0.055) | 77 (<12-582) | <0.06 (<0.06-0.69) | <40 (<40-939) | 0.10 (<0.05-0.79) | <0.06 (<0.06-6.07) | 91 (21-723) | 2.4 (1.1-43.5) |
| Benincasa et al., 2007 | Italy (Calabria, Apulia, Umbria and  Abruzzo) | - | - | - | 89.3-551 | - | - | - | - | 56-1030 | nd-12.8 |
| Camin et al., 2010 | Italy (South: Calabria, Apulia and Sicily; Centre: Lazio, Tuscany and Umbria; North: Veneto and Trentino) | 0.083-0.819 | 0.127-26.3 | - | - | - | 33-9940 | 0.001-2.94 | 0.003-0.208 | 15-495 | 0.058-10.0 |
| Russo et al., 2020 | Italy  (Campania region) | - | 0.98-3.29 | - | 2.90-10.6 | - | 50.5-1200 | - | 0.23-43.5 | 11.5-66.5 | 0.32-1.07 |
| Pošćić et al., 2019 | Croatia | 0.001 (0.001–0.004) | 0.30 (0.06–1.99) | - | 1.11 (0.19–2.57) | - | 286 (7.28–1670) | 0.001 (0.001–0.02) | 0.01 (0.005–0.04) | 24.6 (2.31–99.2) | 0.25 (0.05–7.18) |
| Beltran et al., 2015 | Spain (four  Spanish municipalities of Huelva) | nd-0.2 | 13.9-47.2 | - | 0.5-1.2 | 1.6-4.5 | 5.9-6.9 | - | 2.9-6.4 | 0.6-0.9 | 0.1-0.2 |
| Cabrera-Vique et al., 2012 | Spain (pooled data)  Granada province  Jaén province | - | 3.35-66.47 | - | 25-273 | - | - | - | - | - | 11.6-69.4 |
| Llorent-Martínez et al., 2011(food control) | Spain | - | <8 | - | <40-120 | - | - | - | - | - | - |
| Llorent-Martínez et al., 2014 | Spain | - | 50 | - | <600 | - | - | - | - | - | - |
| Damak et al., 2019 | Tunisia (four geographical  Origins) | - | - | - | 280-570 | - | - | - | - | 850-1200 | 12-18 |
| Gumus et al., 2017 | Turkey | - | 0.112-2.020 | - | 1-14670 | - | 0.000-104 | - | - | 0.000-26 | 0.065-0.910 |

**Table S3.** Element levels [mean or median (range minimum-maximum); μg kg^-1^] in extra-virgin or virgin olive oils from different Mediterranean countries.

| **Reference** | **Country** | **Mo** | **Na** | **Nb** | **Nd** | **Ni** | **P** | **Pb** | **Pr** | **Rb** | **Sb** | **Se** |
| --- | --- | --- | --- | --- | --- | --- | --- | --- | --- | --- | --- | --- |
| **This study** | Italy | <0.3 (<0.3-2.0) | 110 (<25-585) | <0.04 (<0.04-0.11) | 0.03 (<0.03-13.8) | 5.6 (2.1-49.7) | 272 (127-650) | 0.9 (<0.3-22.1) | <0.008 (<0.008-1.65) | 0.24 (<0.06-1.77) | <0.02 (<0.02-0.37) | <0.6 (<0.6-7.8) |
| Benincasa et al., 2007 | Italy (Calabria, Apulia, Umbria and  Abruzzo) | - | - | - | - | nd-46.9 | - | - | - | - | 0.194-0.411 | 1.47-6.78 |
| Camin et al., 2010 | Italy (South: Calabria, Apulia and Sicily; Centre: Lazio, Tuscany and Umbria; North: Veneto and Trentino) | - | 49-609 | - | - | - | - | 0.180-8.46 | - | 0.040-13.4 | - | - |
| Russo et al., 2020 | Italy  (Campania region) | - | 186-830 | - | - | - | 39-148 | 0.76-1.08 | - | - | - | - |
| Pošćić et al., 2019 | Croatia | 0.01 (0.003–0.01) | 4.92 (1.74–315) | - | - | 0.08 (0.05–0.14) | 28.2 (3.83–367) | 0.02 (0.02–0.52) | - | 0.24 (0.01–1.81) | 0.003 (0.002–0.01) | - |
| Beltran et al., 2015 | Spain (four  Spanish municipalities of Huelva) | 13.1-24.3 | 3.8-7.8 | 0.5-1.1 | - | 79.7-173.9 | - | 48.3-74.2 | - | 0.8-2.6 | - | 9.7-13.3 |
| Cabrera-Vique et al., 2012 | Spain (pooled data)  Granada province  Jaén province | - | - | - | - | 10.2-56.8 | - | - | - | - | - | - |
| Llorent-Martínez et al., 2011(food control) | Spain | - | - | - | - | - | - | <0.9 | - | - | - | - |
| Llorent-Martínez et al., 2014 | Spain | - | - | - | - | 130 | - | 50 | - | - | 100 | - |
| Damak et al., 2019 | Tunisia (four geographical  Origins) | - | 1600-4300 | - | - | - | - | 5-7.4 | - | 2.5-3.5 | - | - |
| Gumus et al., 2017 | Turkey | - | 0.023-0.106 | - | - | 0.18-2.59 | - | 0.03-1.31 | - | - | - | - |

**Table S4.** Element levels [mean or median (range minimum-maximum); μg kg^-1^] in extra-virgin or virgin olive oils from different Mediterranean countries.

| **Reference** | **Country** | **Si** | **Sr** | **Tb** | **Te** | **Ti** | **Tl** | **U** | **V** | **W** | **Zn** | **Zr** |
| --- | --- | --- | --- | --- | --- | --- | --- | --- | --- | --- | --- | --- |
| **This study** | Italy | <270 (<270-3340) | 3 (1-58) | <0.006 (<0.006-1.28) | <0.03 (<0.03-0.06) | 1.9 (0.8-10.7) | <0.06 | <0.005 (<0.005-0.050) | 0.53 (<0.08-1.40) | <0.3 (<0.3-5.1) | 111 (54-749) | <0.1 (<0.1-2.3) |
| Benincasa et al., 2007 | Italy (Calabria, Apulia, Umbria and  Abruzzo) | - | nd-48.9 | - | - | - | - | - | - | - | - | - |
| Camin et al., 2010 | Italy (South: Calabria, Apulia and Sicily; Centre: Lazio, Tuscany and Umbria; North: Veneto and Trentino) | - | 1.12-3.85 | - | - | - | - | 0.0008-0.119 | - | - | - | - |
| Russo et al., 2020 | Italy  (Campania region) | - | - | - | - | - | - | - | - | - | 3.44-17.99 | - |
| Pošćić et al., 2019 | Croatia | - | 0.02 (0.01–0.17) | - | - | 0.01 (0.004–0.05) | <0.001 | - | 0.002 (0.002–0.01) | - | 0.88 (0.25–5.61) | - |
| Beltran et al., 2015 | Spain (four  Spanish municipalities of Huelva) | - | nd | - | - | nd | - | 0.1 | 13.8-18.4 | 0.7-4.1 | 149-328 | 3.1-4.3 |
| Llorent-Martínez et al., 2014 | Spain | - | - | - | - | - | - | - | 100 | - | - | - |
| Damak et al., 2019 | Tunisia (four geographical  Origins) | - | 33-37 | - | - | - | - | - | 4.8-5.2 | - | 170-290 | - |
| Gumus et al., 2017 | Turkey | - |  | - | - | - | - | - | 0.00-0.140 | - | 1.14-198 | - |

**Table S5.** Element levels [median, minimum (min) and maximum (max); μg kg^-1^] and antioxidant activity (DPPH%) of EVOOs from northern Italy (Liguria, n=6; Lombardy, n=3; Trentino Alto Adige, n=7; and Veneto, n=3).

| **Element** | **Liguria** | | | | **Lombardy** | | | **Trentino Alto Adige** | | | **Veneto** | | |
| --- | --- | --- | --- | --- | --- | --- | --- | --- | --- | --- | --- | --- | --- |
|  | **median** | **min** | **max** | **median** | | **min** | **max** | **median** | **min** | **max** | **median** | **min** | **max** |
| **Ag** | <0.06 | <0.06 | 0.15 | <0.06 | | <0.06 | 0.26 | <0.06 | <0.06 | 0.13 | <0.06 | <0.06 | <0.06 |
| **Al** | 32 | <9 | 615 | 34 | | <9 | 175 | 21 | <9 | 484 | 39 | 34 | 44 |
| **As** | <0.3 | <0.3 | 0.4 | <0.3 | | <0.3 | 1.1 | <0.3 | <0.3 | 2.8 | <0.3 | <0.3 | 0.6 |
| **B** | <20 | <20 | 85 | <20 | | <20 | <20 | <20 | <20 | 74 | <20 | <20 | <20 |
| **Ba** | 0.9 | <0.7 | 13.0 | <0.7 | | <0.7 | 15.5 | 2.9 | <0.7 | 44.8 | <0.7 | <0.7 | <0.7 |
| **Be** | 0.004 | <0.004 | 0.008 | <0.004 | | <0.004 | 0.007 | 0.005 | <0.004 | 0.431 | <0.004 | <0.004 | <0.004 |
| **Bi** | <0.1 | <0.1 | 0.2 | <0.1 | | <0.1 | 0.1 | 0.1 | <0.1 | 0.1 | <0.1 | <0.1 | 0.1 |
| **Ca** | 4320 | 1840 | 7740 | 3610 | | 1560 | 4430 | 4800 | 1480 | 9170 | 5540 | 4740 | 8890 |
| **Cd** | 0.12 | <0.07 | 0.16 | 0.09 | | <0.07 | 0.16 | 0.17 | <0.07 | 0.33 | 0.12 | 0.10 | 0.14 |
| **Ce** | 0.3 | 0.1 | 0.4 | 0.2 | | 0.1 | 0.5 | 0.2 | 0.1 | 0.7 | 0.2 | 0.1 | 0.3 |
| **Co** | 0.16 | 0.05 | 0.29 | 0.25 | | <0.05 | 0.53 | 0.12 | 0.06 | 0.59 | 0.06 | <0.05 | 0.07 |
| **Cr** | 8 | 1 | 839 | 7 | | 1 | 86 | 6 | 1 | 24 | 3 | 1 | 4 |
| **Cs** | 0.017 | <0.007 | 0.051 | 0.017 | | 0.013 | 0.068 | 0.012 | <0.007 | 0.080 | 0.012 | 0.010 | 0.026 |
| **Cu** | 4.3 | <0.6 | 8.3 | 2.3 | | <0.6 | 4.5 | 6.1 | <0.6 | 20.7 | 7.6 | 4.8 | 11.8 |
| **Dy** | <0.005 | <0.005 | 0.010 | <0.005 | | <0.005 | 0.005 | <0.005 | <0.005 | 0.007 | <0.005 | <0.005 | <0.005 |
| **Fe** | 118 | 62 | 495 | 171 | | 27 | 183 | 136 | <12 | 283 | 218 | 191 | 223 |
| **Ga** | <0.06 | <0.06 | 0.24 | <0.06 | | <0.06 | 0.10 | <0.06 | <0.06 | 0.10 | <0.06 | <0.06 | 0.33 |
| **K** | <40 | <40 | 115 | <40 | | <40 | 116 | <40 | <40 | 293 | 89 | <40 | 154 |
| **La** | 0.16 | <0.05 | 0.22 | 0.17 | | <0.05 | 0.23 | 0.11 | <0.05 | 0.41 | 0.13 | 0.08 | 0.21 |
| **Li** | <0.06 | <0.06 | 0.45 | <0.06 | | <0.06 | 0.53 | <0.06 | <0.06 | 1.67 | <0.06 | <0.06 | 0.16 |
| **Mg** | 89 | 69 | 132 | 95 | | 58 | 103 | 98 | 42 | 262 | 164 | 110 | 171 |
| **Mn** | 2.9 | 2.4 | 6.1 | 2.3 | | 1.8 | 5.0 | 2.1 | 1.5 | 7.1 | 4.4 | 3.0 | 4.8 |
| **Mo** | <0.3 | <0.3 | 1.3 | <0.3 | | <0.3 | 0.7 | <0.3 | <0.3 | 0.6 | <0.3 | <0.3 | <0.3 |
| **Na** | 133 | 95 | 331 | 118 | | 116 | 120 | 169 | 87 | 298 | 174 | 142 | 229 |
| **Nb** | <0.04 | <0.04 | 0.05 | <0.04 | | <0.04 | <0.04 | <0.04 | <0.04 | 0.05 | <0.04 | <0.04 | <0.04 |
| **Nd** | 0.06 | 0.03 | 0.41 | 0.09 | | <0.03 | 0.71 | 0.07 | <0.03 | 1.39 | 0.05 | <0.03 | 0.08 |
| **Ni** | 6.3 | 3.0 | 29.5 | 2.8 | | 2.5 | 6.6 | 5.1 | 3.2 | 13.9 | 5.4 | 4.0 | 6.2 |
| **P** | 330 | 263 | 371 | 281 | | 272 | 360 | 276 | 220 | 650 | 313 | 310 | 317 |
| **Pb** | 1.2 | 0.4 | 2.1 | 1.4 | | 1.0 | 2.1 | 1.0 | 0.1 | 4.3 | 2.1 | 1.7 | 2.2 |
| **Pr** | 0.021 | <0.008 | 0.100 | 0.015 | | 0.009 | 0.173 | 0.012 | <0.008 | 0.359 | <0.008 | <0.008 | 0.012 |
| **Rb** | 0.29 | 0.20 | 0.77 | 0.36 | | 0.29 | 0.76 | 0.24 | 0.14 | 1.10 | 0.37 | 0.36 | 0.39 |
| **Sb** | <0.02 | <0.02 | <0.02 | <0.02 | | <0.02 | 0.04 | <0.02 | <0.02 | 0.04 | <0.02 | <0.02 | <0.02 |
| **Se** | 1.6 | <0.6 | 3.1 | <0.6 | | <0.6 | 5.2 | 0.6 | <0.6 | 6.8 | 0.6 | <0.6 | 3.0 |
| **Si** | <270 | <270 | <270 | <270 | | <270 | <270 | <270 | <270 | <270 | <270 | <270 | <270 |
| **Sn** | 0.10 | 0.06 | 0.45 | 0.06 | | <0.06 | 0.32 | 0.07 | <0.06 | 0.43 | 0.23 | 0.11 | 0.28 |
| **Sr** | 2 | 1 | 4 | 2 | | 1 | 3 | 3 | 1 | 7 | 5 | 3 | 5 |
| **Tb** | <0.006 | <0.006 | 0.022 | <0.006 | | <0.006 | 0.044 | <0.006 | <0.006 | 0.112 | <0.006 | <0.006 | <0.006 |
| **Te** | <0.03 | <0.03 | <0.03 | <0.03 | | <0.03 | <0.03 | <0.03 | <0.03 | <0.03 | <0.03 | <0.03 | 0.05 |
| **Ti** | 1.9 | 1.6 | 8.1 | 2.7 | | 1.6 | 2.8 | 2.4 | 1.6 | 6.2 | 2.2 | 1.5 | 4.7 |
| **Tl** | <0.06 | <0.06 | <0.06 | <0.06 | | <0.06 | <0.06 | <0.06 | <0.06 | <0.06 | <0.06 | <0.06 | <0.06 |
| **U** | 0.009 | <0.005 | 0.020 | 0.006 | | <0.005 | 0.044 | 0.007 | <0.005 | 0.039 | <0.005 | <0.005 | 0.012 |
| **V** | 0.53 | <0.08 | 0.74 | 0.38 | | <0.08 | 0.59 | 0.47 | 0.34 | 1.04 | 0.54 | 0.53 | 0.64 |
| **W** | <0.3 | <0.3 | 1.5 | 0.3 | | <0.3 | 1.1 | <0.3 | <0.3 | <0.3 | <0.3 | <0.3 | <0.3 |
| **Zn** | 111 | 63 | 236 | 90 | | 56 | 163 | 185 | 55 | 258 | 184 | 171 | 283 |
| **Zr** | 0.2 | 0.1 | 0.6 | 0.2 | | 0.1 | 0.3 | 0.1 | 0.1 | 0.5 | 0.2 | 0.1 | 0.3 |
| **%DPPH** | 15.7 | 9.2 | 20.7 | 22.6 | | 19.2 | 26.0 | 24.3 | 21.0 | 36.4 | 14.8 | 12.8 | 22.2 |

**Table S6.** Element levels [median, minimum (min) and maximum (max); μg kg^-1^] and antioxidant activity (DPPH%) of EVOOs from central Italy (Abruzzo, n=14; Lazio, n=24; Marche, n=7; Tuscany, n=79; and Umbria, n=8).

| **Element** | **Abruzzo** | | | **Lazio** | | | **Marche** | | | **Tuscany** | | | **Umbria** | | |
| --- | --- | --- | --- | --- | --- | --- | --- | --- | --- | --- | --- | --- | --- | --- | --- |
|  | **median** | **min** | **max** | **median** | **min** | **max** | **median** | **min** | **max** | **median** | **min** | **max** | **median** | **min** | **max** |
| **Ag** | <0.06 | <0.06 | 0.10 | <0.06 | 0.03 | 0.86 | <0.06 | <0.06 | 0.07 | <0.06 | <0.06 | 0.20 | <0.06 | <0.06 | 0.06 |
| **Al** | 33 | <9 | 347 | 45 | <9 | 606 | 31 | 27 | 78 | 34 | <9 | 1291 | 29 | <9 | 66 |
| **As** | <0.3 | <0.3 | <0.3 | <0.3 | <0.3 | 1.9 | <0.3 | <0.3 | <0.3 | <0.3 | <0.3 | 4.0 | <0.3 | <0.3 | 1.2 |
| **B** | <20 | <20 | <20 | <20 | <20 | 530 | <20 | <20 | <20 | <20 | <20 | 734 | <20 | <20 | <20 |
| **Ba** | 7.1 | <0.7 | 175.1 | <0.7 | <0.7 | 36.8 | 37.9 | 3.6 | 110.8 | 3.2 | <0.7 | 170.2 | 4.0 | <0.7 | 63.6 |
| **Be** | <0.004 | <0.004 | 0.015 | <0.004 | <0.004 | 0.034 | <0.004 | <0.004 | <0.004 | <0.004 | <0.004 | 0.272 | <0.004 | <0.004 | 0.008 |
| **Bi** | <0.1 | <0.1 | 0.1 | <0.1 | <0.1 | 0.3 | <0.1 | <0.1 | <0.1 | <0.1 | <0.1 | 0.4 | <0.1 | <0.1 | 0.1 |
| **Ca** | 1880 | 1230 | 1880 | 4660 | 1600 | 35700 | 2140 | 1600 | 2890 | 3630 | 1330 | 14700 | 4370 | 1500 | 5550 |
| **Cd** | <0.07 | <0.07 | 0.12 | 0.14 | <0.07 | 0.46 | <0.07 | <0.07 | <0.07 | 0.08 | <0.07 | 0.97 | 0.09 | <0.07 | 0.23 |
| **Ce** | 0.1 | 0.1 | 0.4 | 0.3 | 0.1 | 3.5 | 0.1 | 0.1 | 0.1 | 0.2 | 0.1 | 2.5 | 0.1 | 0.1 | 0.8 |
| **Co** | 0.17 | 0.12 | 0.87 | 0.18 | <0.05 | 1.23 | 0.12 | 0.07 | 0.16 | 0.10 | <0.05 | 0.71 | <0.05 | <0.05 | 0.39 |
| **Cr** | 7.7 | 3.7 | 27.8 | 6.6 | 0.6 | 123 | 5.2 | 3.7 | 9.3 | 4.0 | <0.3 | 42.6 | 2.1 | 0.7 | 35.4 |
| **Cs** | 0.007 | <0.007 | 0.048 | 0.023 | <0.007 | 0.084 | <0.007 | <0.007 | <0.007 | <0.007 | <0.007 | 0.052 | 0.008 | <0.007 | 0.020 |
| **Cu** | 2.9 | <0.6 | 23.0 | 4.2 | <0.6 | 18.1 | 2.8 | 2.0 | 11.3 | 2.9 | <0.6 | 40.9 | 2.6 | <0.6 | 4.3 |
| **Dy** | <0.005 | <0.005 | <0.005 | 0.007 | <0.005 | 0.026 | <0.005 | <0.005 | <0.005 | <0.005 | <0.005 | 0.011 | <0.005 | <0.005 | <0.005 |
| **Fe** | 15 | <12 | 130 | 103 | <12 | 302 | 18 | 12 | 30 | 70 | <12 | 403 | 156 | 46 | 263 |
| **Ga** | <0.06 | <0.06 | <0.06 | <0.06 | <0.06 | 0.69 | <0.06 | <0.06 | <0.06 | <0.06 | <0.06 | 0.44 | <0.06 | <0.06 | <0.06 |
| **K** | <40 | <40 | 140 | <40 | <40 | 164 | <40 | <40 | <40 | <40 | <40 | 673 | <40 | <40 | 97 |
| **La** | <0.05 | <0.05 | 0.17 | 0.20 | <0.05 | 0.79 | <0.05 | <0.05 | 0.06 | 0.09 | <0.05 | 0.30 | 0.08 | <0.05 | 0.58 |
| **Li** | 0.06 | <0.06 | 0.65 | 0.13 | <0.06 | 6.07 | <0.06 | <0.06 | 0.13 | <0.06 | <0.06 | 2.51 | <0.06 | <0.06 | 0.27 |
| **Mg** | 56 | 35 | 385 | 115 | 46 | 723 | 48 | 28 | 90 | 89 | 21 | 307 | 93 | 59 | 122 |
| **Mn** | 2.2 | 1.5 | 10.8 | 4.1 | 1.5 | 8.7 | 1.9 | 1.4 | 2.4 | 2.2 | 1.1 | 18.6 | 2.3 | 1.6 | 3.5 |
| **Mo** | <0.3 | <0.3 | 0.3 | <0.3 | <0.3 | 0.9 | <0.3 | <0.3 | <0.3 | <0.3 | <0.3 | 1.7 | <0.3 | <0.3 | <0.3 |
| **Na** | 114 | <25 | 288 | 136 | 81 | 585 | 38 | <25 | 99 | 97 | 40 | 341 | 96 | 79 | 273 |
| **Nb** | <0.04 | <0.04 | <0.04 | <0.04 | <0.04 | 0.06 | <0.04 | <0.04 | <0.04 | <0.04 | <0.04 | 0.05 | <0.04 | <0.04 | <0.04 |
| **Nd** | <0.03 | <0.03 | 0.12 | 0.13 | <0.03 | 6.43 | <0.03 | <0.03 | <0.03 | <0.03 | <0.03 | 3.12 | 0.03 | <0.03 | 0.10 |
| **Ni** | 9.3 | 5.4 | 36.1 | 6.0 | 3.2 | 20.0 | 7.9 | 5.5 | 9.8 | 5.0 | 2.1 | 40.6 | 6.4 | 3.1 | 16.2 |
| **P** | 270 | 254 | 387 | 285 | 163 | 479 | 275 | 255 | 294 | 265 | 127 | 522 | 283 | 203 | 332 |
| **Pb** | 0.5 | <0.3 | 6.4 | 1.5 | <0.3 | 4.4 | 0.4 | 0.3 | 0.6 | 0.8 | <0.3 | 22.1 | 0.8 | <0.3 | 1.6 |
| **Pr** | <0.008 | <0.008 | 0.013 | 0.030 | <0.008 | 1.582 | <0.008 | <0.008 | <0.008 | <0.008 | <0.008 | 0.773 | 0.006 | <0.008 | 0.020 |
| **Rb** | 0.20 | 0.09 | 0.51 | 0.48 | 0.07 | 1.77 | 0.10 | 0.06 | 0.17 | 0.23 | 0.08 | 0.83 | 0.26 | 0.13 | 0.38 |
| **Sb** | <0.02 | <0.02 | 0.17 | <0.02 | <0.02 | 0.37 | <0.02 | <0.02 | <0.02 | <0.02 | <0.02 | 0.17 | <0.02 | <0.02 | <0.02 |
| **Se** | <0.6 | <0.6 | <0.6 | 0.9 | <0.6 | 5.3 | <0.6 | <0.6 | <0.6 | <0.6 | <0.6 | 6.9 | 1.8 | <0.6 | 5.9 |
| **Si** | <270 | <270 | <270 | <270 | <270 | <270 | <270 | <270 | <270 | <270 | <270 | 3344 | <270 | <270 | <270 |
| **Sn** | <0.06 | <0.06 | 0.29 | 0.12 | <0.06 | 0.60 | <0.06 | <0.06 | 0.13 | 0.06 | <0.06 | 0.50 | <0.06 | <0.06 | 0.14 |
| **Sr** | 1 | 1 | 34 | 4 | 1 | 23 | 1 | 1 | 4 | 3 | 1 | 19 | 3 | 1 | 7 |
| **Tb** | <0.006 | <0.006 | 0.006 | <0.006 | 0.003 | 1.133 | <0.006 | <0.006 | <0.006 | <0.006 | <0.006 | 0.237 | <0.006 | <0.006 | 0.006 |
| **Te** | <0.03 | <0.03 | 0.05 | <0.03 | <0.03 | <0.03 | <0.03 | <0.03 | <0.03 | <0.03 | <0.03 | 0.03 | <0.03 | <0.03 | <0.03 |
| **Ti** | 1.6 | 1.3 | 3.5 | 2.7 | 1.2 | 5.6 | 1.5 | 1.2 | 2.0 | 1.7 | 0.8 | 4.2 | 1.9 | 1.2 | 2.8 |
| **Tl** | <0.06 | <0.06 | <0.06 | <0.06 | <0.06 | 0.08 | <0.06 | <0.06 | <0.06 | <0.06 | <0.06 | <0.06 | <0.06 | <0.06 | <0.06 |
| **U** | <0.005 | <0.005 | 0.008 | 0.012 | <0.005 | 0.044 | <0.005 | <0.005 | <0.005 | <0.005 | <0.005 | 0.027 | <0.005 | <0.005 | 0.006 |
| **V** | 0.39 | 0.13 | 0.70 | 0.60 | 0.15 | 1.06 | 0.32 | 0.25 | 0.52 | 0.54 | <0.08 | 1.21 | 0.58 | 0.15 | 0.80 |
| **W** | <0.3 | <0.3 | 1.0 | 0.5 | <0.3 | 1.8 | <0.3 | <0.3 | <0.3 | <0.3 | <0.3 | 2.3 | <0.3 | <0.3 | 0.3 |
| **Zn** | 68 | 58 | 177 | 150 | 60 | 749 | 75 | 61 | 97 | 98 | 54 | 409 | 147 | 60 | 189 |
| **Zr** | 0.1 | 0.1 | 0.7 | 0.2 | 0.1 | 1.6 | 0.1 | 0.1 | 0.7 | 0.1 | 0.1 | 1.8 | 0.1 | 0.1 | 0.2 |
| **%DPPH** | 47.0 | 19.7 | 63.1 | 31.6 | 8.0 | 49.2 | 34.7 | 23.6 | 39.0 | 36.2 | 9.4 | 66.0 | 29.8 | 27.4 | 36.8 |

**Table S7.** Element levels [median, minimum (min) and maximum (max); μg kg^-1^] and antioxidant activity (DPPH%) of EVOOs from southern Italy (Calabria, n=12; Campania, n=7; Apulia, n=33; Sardinia, n=12; and Sicily, n=21).

| **Element** | **Calabria** | | | **Campania** | | | **Apulia** | | | **Sardinia** | | | **Sicily** | | |
| --- | --- | --- | --- | --- | --- | --- | --- | --- | --- | --- | --- | --- | --- | --- | --- |
|  | **median** | **min** | **max** | **median** | **min** | **max** | **median** | **min** | **max** | **median** | **min** | **max** | **median** | **min** | **max** |
| **Ag** | <0.06 | <0.06 | 0.11 | <0.06 | <0.06 | <0.06 | <0.06 | <0.06 | 0.22 | <0.06 | <0.06 | 0.15 | <0.06 | <0.06 | 0.22 |
| **Al** | 33 | 15 | 694 | 35 | <9 | 43 | 53 | 23 | 1298 | 21 | <9 | 36 | 33 | <9 | 323 |
| **As** | <0.3 | <0.3 | 0.8 | <0.3 | <0.3 | <0.3 | <0.3 | <0.3 | 1.0 | <0.3 | <0.3 | 1.8 | 0.6 | <0.3 | 2.2 |
| **B** | <20 | <20 | 425 | <20 | <20 | <20 | <20 | <20 | 770 | <20 | <20 | 245 | <20 | <20 | 182 |
| **Ba** | <0.7 | <0.7 | 33.1 | 17.5 | 3.9 | 86.9 | <0.7 | <0.7 | 147.1 | 2.8 | <0.7 | 42.2 | <0.7 | <0.7 | 123.4 |
| **Be** | <0.004 | <0.004 | 0.017 | <0.004 | <0.004 | 0.006 | 0.004 | <0.004 | 0.061 | <0.004 | <0.004 | 0.011 | 0.013 | <0.004 | 0.048 |
| **Bi** | <0.1 | <0.1 | 0.2 | <0.1 | <0.1 | 0.4 | <0.1 | <0.1 | 0.2 | <0.1 | <0.1 | 0.1 | <0.1 | <0.1 | 1.0 |
| **Ca** | 4830 | 2040 | 11500 | 2050 | 1430 | 2610 | 4090 | 1640 | 24100 | 4210 | 1700 | 6790 | 4980 | 1480 | 8590 |
| **Cd** | 0.12 | <0.07 | 0.31 | <0.07 | <0.07 | 0.09 | 0.11 | <0.07 | 0.61 | 0.09 | <0.07 | 0.21 | 0.10 | <0.07 | 0.41 |
| **Ce** | 0.2 | 0.1 | 1.0 | 0.1 | 0.1 | 0.1 | 0.2 | 0.1 | 0.9 | 0.1 | 0.1 | 0.2 | 0.2 | 0.1 | 1.0 |
| **Co** | 0.09 | <0.05 | 2.16 | 0.22 | 0.06 | 0.63 | 0.08 | <0.05 | 1.68 | 0.16 | <0.05 | 0.27 | <0.05 | <0.05 | 0.79 |
| **Cr** | 5.2 | 0.5 | 532.7 | 6.0 | 3.6 | 17.8 | 3.4 | 1.0 | 154.1 | 5.1 | 0.5 | 15.8 | 3.7 | 1.1 | 251.9 |
| **Cs** | 0.010 | <0.007 | 0.060 | <0.007 | <0.007 | 0.023 | 0.008 | <0.007 | 0.101 | 0.010 | <0.007 | 0.025 | 0.007 | <0.007 | 0.071 |
| **Cu** | 3.9 | <0.6 | 18.7 | 3.5 | 2.0 | 41.6 | 3.9 | 2.0 | 28.0 | 1.4 | <0.6 | 6.0 | 3.2 | 1.8 | 21.9 |
| **Dy** | <0.005 | <0.005 | 0.013 | <0.005 | <0.005 | <0.005 | <0.005 | <0.005 | 0.032 | <0.005 | <0.005 | <0.005 | <0.005 | <0.005 | 0.055 |
| **Fe** | 147 | 35 | 262 | 24 | 15 | 38 | 74 | 14 | 197 | 99 | 19 | 193 | 121 | 17 | 582 |
| **Ga** | <0.06 | <0.06 | 0.27 | <0.06 | <0.06 | <0.06 | <0.06 | <0.06 | 0.35 | <0.06 | <0.06 | <0.06 | <0.06 | <0.06 | 0.59 |
| **K** | <40 | <40 | 63 | <40 | <40 | 80 | <40 | <40 | 939 | <40 | <40 | 138 | <40 | <40 | 479 |
| **La** | 0.11 | <0.05 | 0.38 | <0.05 | <0.05 | <0.05 | 0.12 | <0.05 | 0.56 | 0.06 | <0.05 | 0.13 | 0.13 | <0.05 | 0.71 |
| **Li** | <0.06 | <0.06 | 0.32 | <0.06 | <0.06 | 0.29 | <0.06 | <0.06 | 4.42 | <0.06 | <0.06 | 0.23 | <0.06 | <0.06 | 1.07 |
| **Mg** | 104 | 58 | 223 | 51 | 38 | 61 | 102 | 33 | 613 | 86 | 42 | 155 | 98 | 28 | 152 |
| **Mn** | 2.7 | 2.1 | 11.6 | 2.7 | 1.7 | 5.4 | 3.0 | 1.4 | 10.2 | 2.2 | 1.7 | 3.6 | 2.6 | 1.5 | 43.5 |
| **Mo** | <0.3 | <0.3 | 0.9 | <0.3 | <0.3 | 0.4 | <0.3 | <0.3 | 2.0 | <0.3 | <0.3 | <0.3 | <0.3 | <0.3 | 0.7 |
| **Na** | 134 | 92 | 458 | 65 | <25 | 92 | 117 | <25 | 390 | 141 | 91 | 229 | 102 | 35 | 513 |
| **Nb** | <0.04 | <0.04 | 0.05 | <0.04 | <0.04 | <0.04 | <0.04 | <0.04 | 0.11 | <0.04 | <0.04 | <0.04 | <0.04 | <0.04 | 0.06 |
| **Nd** | 0.05 | <0.03 | 0.63 | <0.03 | <0.03 | 0.17 | 0.03 | <0.03 | 13.83 | <0.03 | <0.03 | 0.07 | 0.03 | <0.03 | 6.77 |
| **Ni** | 6.8 | 3.1 | 9.6 | 8.4 | 5.5 | 27.0 | 4.5 | 2.4 | 49.7 | 6.4 | 3.1 | 9.8 | 4.2 | 2.7 | 19.1 |
| **P** | 296 | 206 | 358 | 271 | 250 | 336 | 268 | 189 | 380 | 293 | 218 | 383 | 266 | 206 | 548 |
| **Pb** | 1.2 | <0.3 | 3.4 | 0.4 | <0.3 | 1.6 | 1.1 | 0.3 | 7.4 | 0.7 | <0.3 | 1.4 | 1.3 | <0.3 | 8.7 |
| **Pr** | <0.008 | <0.008 | 0.152 | <0.008 | <0.008 | 0.011 | <0.008 | <0.008 | 0.681 | <0.008 | <0.008 | 0.012 | <0.008 | <0.008 | 1.653 |
| **Rb** | 0.24 | 0.18 | 0.59 | 0.17 | 0.06 | 0.25 | 0.27 | <0.06 | 1.23 | 0.24 | 0.13 | 0.55 | 0.26 | 0.08 | 1.36 |
| **Sb** | <0.02 | <0.02 | 0.12 | <0.02 | <0.02 | <0.02 | <0.02 | <0.02 | 0.14 | <0.02 | <0.02 | 0.03 | <0.02 | <0.02 | 0.03 |
| **Se** | 1.2 | <0.6 | 3.6 | <0.6 | <0.6 | <0.6 | 0.6 | <0.6 | 5.8 | 0.8 | <0.6 | 4.1 | 1.5 | <0.6 | 7.8 |
| **Si** | <270 | <270 | <270 | <270 | <270 | <270 | <270 | <270 | <270 | <270 | <270 | 442 | <270 | <270 | <270 |
| **Sn** | 0.12 | <0.06 | 0.29 | <0.06 | <0.06 | 0.40 | 0.14 | <0.06 | 1.94 | 0.06 | <0.06 | 0.48 | 0.07 | <0.06 | 0.79 |
| **Sr** | 4 | 1 | 7 | 1 | 1 | 1 | 3 | 1 | 58 | 2 | 1 | 7 | 4 | 1 | 52 |
| **Tb** | <0.006 | <0.006 | 0.039 | <0.006 | <0.006 | <0.006 | <0.006 | <0.006 | 1.279 | <0.006 | <0.006 | <0.006 | <0.006 | <0.006 | 0.504 |
| **Te** | <0.03 | <0.03 | <0.03 | <0.03 | <0.03 | <0.03 | <0.03 | <0.03 | 0.03 | <0.03 | <0.03 | <0.03 | <0.03 | <0.03 | 0.06 |
| **Ti** | 1.9 | 1.3 | 4.9 | 1.5 | 1.2 | 10.7 | 2.9 | 1.1 | 4.6 | 1.9 | 1.3 | 2.5 | 2.4 | 1.4 | 4.5 |
| **Tl** | <0.06 | <0.06 | <0.06 | <0.06 | <0.06 | <0.06 | <0.06 | <0.06 | <0.06 | <0.06 | <0.06 | <0.06 | <0.06 | <0.06 | <0.06 |
| **U** | <0.005 | <0.005 | 0.029 | <0.005 | <0.005 | <0.005 | <0.005 | <0.005 | 0.050 | <0.005 | <0.005 | 0.005 | <0.005 | <0.005 | 0.050 |
| **V** | 0.52 | 0.04 | 0.80 | 0.30 | <0.08 | 0.47 | 0.67 | <0.08 | 1.40 | 0.49 | 0.40 | 0.76 | 0.74 | <0.08 | 0.88 |
| **W** | <0.3 | <0.3 | 1.0 | <0.3 | <0.3 | <0.3 | <0.3 | <0.3 | 5.1 | <0.3 | <0.3 | 0.7 | <0.3 | <0.3 | 2.1 |
| **Zn** | 147 | 59 | 346 | 67 | 58 | 95 | 114 | 61 | 672 | 151 | 59 | 210 | 167 | 57 | 261 |
| **Zr** | 0.1 | 0.1 | 0.5 | 0.1 | 0.1 | 0.1 | 0.2 | 0.1 | 2.3 | 0.1 | 0.1 | 0.1 | 0.1 | 0.1 | 0.7 |
| **%DPPH** | 22.9 | 10.9 | 43.1 | 28.3 | 19.1 | 67.3 | 37.6 | 14.4 | 53.1 | 28.0 | 14.2 | 40.7 | 18.2 | 8.3 | 45.3 |

**Table S8.** Spearman’s correlation coefficients of the antioxidant activity (DPPH%) and elements of EVOOs^a^.

|  | **DPPH%** | **Region** | | | | | | |
| --- | --- | --- | --- | --- | --- | --- | --- | --- |
| **Elements** | **All samples** | **Abruzzo** | **Lazio** | **Tuscany** | **Calabria** | **Apulia** | **Sardinia** | **Sicily** |
| **Ag** | -0.041 | 0.126 | -0.261 | 0.133 | -0.082 | -0.342 | 0.044 | -0.105 |
| **Al** | 0.031 | 0.553* | -0.279 | 0.133 | -0.467 | -0.290 | 0.500 | -0.483* |
| **As** | -0.231** | nd^b^ | -0.327 | -0.355** | 0.548 | -0.343 | 0.560 | -0.220 |
| **B** | -0.159* | nd^b^ | 0.189 | -0.200 | -0.548 | -0.332 | 0.624* | -0.268 |
| **Ba** | 0.336** | 0.371 | 0.129 | 0.336** | 0.495 | 0.500* | -0.247 | 0.182 |
| **Be** | -0.236** | -0.574* | -0.281 | -0.218 | -0.037 | -0.008 | -0.239 | -0.015 |
| **Bi** | -0.130 | -0.224 | -0.364 | -0.071 | -0.342 | -0.334 | 0.480 | -0.490* |
| **Ca** | -0.305** | 0.534* | -0.182 | -0.350** | -0.683* | -0.492* | 0.343 | -0.341 |
| **Cd** | -0.340** | -0.256 | -0.226 | -0.409** | -0.343 | -0.548* | 0.329 | -0.273 |
| **Ce** | -0.371** | 0.094 | -0.455* | -0.453** | -0.583 | -0.439* | 0.537 | -0.635** |
| **Co** | 0.016 | 0.213 | -0.504* | 0.100 | 0.059 | -0.005 | -0.513 | -0.199 |
| **Cr** | -0.007 | 0.130 | -0.340 | 0.041 | -0.050 | -0.005 | -0.378 | -0.083 |
| **Cs** | -0.319** | -0.505 | -0.486* | -0.272* | 0.059 | -0.437* | -0.253 | -0.464 |
| **Cu** | 0.047 | 0.458 | -0.016 | 0.076 | -0.390 | 0.074 | 0.544 | -0.355 |
| **Dy** | -0.194** | nd^b^ | -0.561** | -0.133 | -0.059 | -0.212 | nd^b^ | -0.497* |
| **Fe** | -0.360** | 0.569* | -0.039 | -0.398** | -0.550 | -0.377 | 0.490 | -0.287 |
| **Ga** | -0.222** | nd^b^ | -0.306 | -0.050 | -0.342 | -0.296 | nd^b^ | -0.490* |
| **K** | -0.084 | 0.366 | -0.414* | -0.082 | 0.411 | -0.007 | 0.184 | -0.385 |
| **La** | -0.430** | -0.351 | -0.458* | -0.518** | -0.500 | -0.497* | 0.466 | -0.620** |
| **Li** | -0.095 | 0.240 | -0.294 | -0.161 | -0.018 | -0.376 | -0.100 | -0.144 |
| **Mg** | -0.355** | 0.213 | -0.399 | -0.402** | -0.583 | -0.581** | 0.420 | -0.441 |
| **Mn** | -0.268** | 0.240 | -0.470* | -0.264* | -0.617 | -0.256 | 0.671* | -0.615** |
| **Mo** | -0.144* | 0.103 | -0.330 | -0.026 | -0.342 | -0.270 | nd^b^ | 0.063 |
| **Na** | -0.280** | -0.059 | -0.231 | -0.305** | -0.383 | -0.559** | -0.629* | -0.213 |
| **Nb** | -0.140* | nd^b^ | -0.160 | 0.023 | -0.342 | -0.209 | nd^b^ | 0.053 |
| **Nd** | -0.341** | -0.297 | -0.548** | -0.322** | -0.033 | -0.308 | 0.146 | -0.490* |
| **Ni** | 0.141* | 0.218 | -0.298 | 0.230* | -0.100 | 0.248 | -0.448 | 0.292 |
| **P** | -0.191** | -0.108 | -0.621** | -0.093 | 0.083 | 0.034 | -0.266 | -0.365 |
| **Pb** | -0.257** | 0.389 | -0.235 | -0.257* | -0.717* | -0.412 | 0.521 | -0.564* |
| **Pr** | -0.315** | -0.750** | -0.602** | -0.251* | 0.044 | -0.398 | -0.516 | -0.576* |
| **Rb** | -0.321** | -0.024 | -0.450* | -0.292** | -0.433 | -0.506* | 0.357 | -0.574* |
| **Sb** | -0.057 | 0.321 | -0.540** | -0.093 | 0.274 | -0.210 | 0.131 | 0.211 |
| **Se** | -0.264** | nd^b^ | 0.135 | -0.367** | -0.164 | -0.389 | 0.616* | -0.127 |
| **Si** | 0.019 | nd^b^ | nd^b^ | 0.073 | nd^b^ | nd^b^ | -0.218 | nd |
| **Sn** | -0.187** | -0.023 | -0.295 | -0.083 | -0.700* | -0.233 | -0.203 | -0.437 |
| **Sr** | -0.294** | 0.260 | -0.350 | -0.316** | -0.109 | -0.252 | 0.537 | -0.420 |
| **Tb** | -0.231** | -0.310 | -0.459* | -0.198 | -0.342 | -0.274 | nd^b^ | -0.512* |
| **Te** | -0.183** | -0.447 | nd | -0.084 | nd^b^ | nd^b^ | nd^b^ | -0.158 |
| **Ti** | -0.342** | -0.015 | -0.457* | -0.471** | -0.600 | -0.410 | 0.399 | -0.792** |
| **Tl** | 0.020 | nd^b^ | 0.105 | nd^b^ | nd^b^ | nd^b^ | nd^b^ | nd^b^ |
| **U** | -0.312** | -0.713** | -0.546** | -0.292** | -0.129 | -0.416 | -0.044 | -0.349 |
| **V** | -0.090 | 0.543* | 0.069 | -0.193 | 0.467 | -0.225 | 0.678* | -0.228 |
| **W** | -0.111 | -0.594* | -0.323 | -0.065 | 0.000 | -0.234 | -0.367 | 0.093 |
| **Zn** | -0.281** | 0.503 | -0.075 | -0.355** | -0.433 | -0.440* | 0.315 | -0.233 |
| **Zr** | -0.194** | 0.768** | -0.264 | -0.081 | -0.661 | -0.438* | 0.437 | -0.578* |

* Correlation is significant at the 0.05 level (two-tailed). ** Correlation is significant at the 0.01 level (two-tailed).

^a^ Only regions with at least 10 EVOO samples were considered.

^b^ nd, not determined.

# References

Beltrán, M., Sánchez-Astudillo, M., Aparicio, R., and García-González, D. L. (2015). Geographical traceability of virgin olive oils from south-western Spain by their multi-elemental composition. *Food Chem.* 169, 350–357. doi: 10.1016/j.foodchem.2014.07.104.

Benincasa, C., Lewis, J., Perri, E., Sindona, G., and Tagarelli, A. (2007). Determination of trace element in Italian virgin olive oils and their characterization according to geographical origin by statistical analysis. *Anal. Chim. Acta* 585, 366–370. doi: 10.1016/j.aca.2006.12.040.

Cabrera-Vique, C., Bouzas, P.R., Oliveras-López, M.J. (2012). Determination of trace elements in extra virgin olive oils: A pilot study on the geographical characterisation, Food Chem. 134(1), 434–439. doi: 10.1016/j.foodchem.2012.02.088.

Camin, F., Larcher, R., Perini, M., Bontempo, L., Bertoldi, D., Gagliano, G., Nicolini, G., and Versini, G. (2010). Characterisation of authentic Italian extra-virgin olive oils by stable isotope ratios of C, O and H and mineral composition. *Food Chem.* 118, 901–909. doi: 10.1016/j.foodchem.2008.04.059.

Damak, F., Asano, M., Baba, K., Suda, A., Araoka, D., Wali, A., Isoda. H., Nakajima, M., Ksibi, M., and Tamura, K. (2019). Interregional traceability of Tunisian olive oils to the provenance soil by multielemental fingerprinting and chemometrics. *Food Chem.* 283, 656–664. doi: 10.1016/j.foodchem.2019.01.082.

Gumus, Z.P., Celenk, V. U., Tekin, S., Yurdakul, O., and Ertas, H. (2017). Determination of trace elements and stable carbon isotope ratios in virgin olive oils from Western Turkey to authenticate geographical origin with a chemometric approach. *Eur. Food Res. Technol.* 243, 1719–1727. doi: 10.1007/s00217-017-2876-4.

Llorent-Martínez, E. J., Ortega-Barrales, P., Fernández-de Córdova, M. L., and Ruiz-Medina, A. (2011). Analysis of the legislated metals in different categories of olive and olive-pomace oils. *Food Control* 22, 221–225. doi: 10.1016/j.foodcont.2010.07.002.

Llorent-Martinez, E. J., Fernandez-de Cordova, M. L., Ortega-Barrales, P., and Ruiz-Medina, A. (2014). Quantitation of metals during the extraction of virgin olive oil from olives using ICP-MS after microwave-assisted acid digestion. *J. Am. Oil Chem. Soc.* 91, 1823–1830. doi: 10.1007/s11746-014-2511-5.

Pošćić, F., Turk, M. F., Bačić, N., Mikac, N., Bertoldi, D., Camin, F., Špika, M. J., Žanetić, M., Rengel, Z., and Perica, S. (2019). Removal of pomace residues is critical in quantification of element concentrations in extra virgin olive oil. *J. Food Compos. Anal.* 77, 39–46. doi: 10.1016/j.jfca.2019.01.002.

Russo, G., Beritognolo, I., Bufacchi, M., Stanzione, V., Pisanelli, A., Ciolfi, M., Lauteri, M., and Brush, S. B. (2020). Advances in biocultural geography of olive tree (Olea europaea L.) landscapes by merging biological and historical assays. *Sci. Rep.* 10, 7673. doi: 10.1038/s41598-020-64063-8
